# Mouse Ifit1b is a cap1-RNA–binding protein that inhibits mouse coronavirus translation and is regulated by complexing with Ifit1c

**DOI:** 10.1074/jbc.RA120.014695

**Published:** 2020-10-19

**Authors:** Harriet V. Mears, Trevor R. Sweeney

**Affiliations:** Division of Virology, Department of Pathology, University of Cambridge, Addenbrooke's Hospital, Cambridge, United Kingdom

**Keywords:** RNA-binding protein, mRNA, translation control, innate immunity, viral immunology, mouse, interferon-induced proteins with tetratricopeptide repeats (IFIT)

## Abstract

Knockout mouse models have been extensively used to study the antiviral activity of IFIT (interferon-induced protein with tetratricopeptide repeats). Human IFIT1 binds to cap0 (m7GpppN) RNA, which lacks methylation on the first and second cap-proximal nucleotides (cap1, m7GpppNm, and cap2, m7GpppNmNm, respectively). These modifications are signatures of “self” in higher eukaryotes, whereas unmodified cap0-RNA is recognized as foreign and, therefore, potentially harmful to the host cell. IFIT1 inhibits translation at the initiation stage by competing with the cap-binding initiation factor complex, eIF4F, restricting infection by certain viruses that possess “nonself” cap0-mRNAs. However, in mice and other rodents, the IFIT1 orthologue has been lost, and the closely related Ifit1b has been duplicated twice, yielding three paralogues: Ifit1, Ifit1b, and Ifit1c. Although murine Ifit1 is similar to human IFIT1 in its cap0-RNA–binding selectivity, the roles of Ifit1b and Ifit1c are unknown. Here, we found that Ifit1b preferentially binds to cap1-RNA, whereas binding is much weaker to cap0- and cap2-RNA. In murine cells, we show that Ifit1b can modulate host translation and restrict WT mouse coronavirus infection. We found that Ifit1c acts as a stimulatory cofactor for both Ifit1 and Ifit1b, promoting their translation inhibition. In this way, Ifit1c acts in an analogous fashion to human IFIT3, which is a cofactor to human IFIT1. This work clarifies similarities and differences between the human and murine IFIT families to facilitate better design and interpretation of mouse models of human infection and sheds light on the evolutionary plasticity of the IFIT family.

Viruses with capped positive-sense RNA genomes must convincingly mimic host mRNA to avoid recognition by cell-intrinsic defense systems. In eukaryotes, the mRNA cap consists of a guanosine nucleotide covalently linked to the first RNA nucleotide by a 5′-5′ triphosphate bridge (capG, GpppNN), which is methylated at the N-7 position (cap0, m^7^GpppNN) to facilitate nuclear export and translation initiation factor recruitment. In higher eukaryotes, including insects and vertebrates, mRNA is further modified by methylation on the 2′-hydroxyl of the first and second cap-proximal nucleotide riboses (cap1, m^7^GpppNmN and cap2, m^7^GpppNmNm) (1). Coronaviruses, including severe acute respiratory syndrome–CoV and the newly emerged severe acute respiratory syndrome–CoV-2, and mosquito-borne flaviviruses, including dengue virus and Zika virus (ZIKV), encode viral 2′-*O*-methyltransferases to produce cap1 viral mRNAs (2), which effectively mimic those of the host to avoid immune surveillance.

Sensing of mRNA 2′-*O*-methylation in vertebrates is primarily mediated by a family of antiviral RNA-binding proteins known as IFITs (interferon-induced proteins with tetratricopeptide repeats). In most mammals, including humans, the IFIT family comprises five members: IFIT1, IFIT1B, IFIT2, IFIT3, and IFIT5 (3, 4). IFITs are comprised of tandem tetratricopeptide repeat motifs that form superhelical N- and C-terminal domains joined by a pivot domain of variable length and flexibility (5). Structures of human IFIT1 and IFIT5 have shown that the groove between these N- and C-terminal domains is lined with positively charged residues which nonspecifically coordinate the phosphate backbone of single-stranded RNA (6–10). As such, IFIT5 binds specifically to single-stranded RNA that lacks a 5′ cap (5′ppp) (7, 11, 12). IFIT1 has an additional hydrophobic cavity within the N terminus that can accommodate the 5′ cap and consequently has high affinity for cap0 RNA (8). However, because of steric restrictions within the mRNA-binding channel, IFIT1 binds to cap1 RNA with much lower affinity and cannot bind to cap2-RNA (8, 13). Binding to the 5′ extremity of cap0 transcripts allows IFIT1 to effectively out-compete the cap-binding eukaryotic translation initiation factor (eIF) 4F, thereby inhibiting translation at the initiation stage (11). Although IFIT3 does not bind RNA directly, its ability to form a complex with IFIT1 via a conserved C-terminal interaction motif greatly increases IFIT1 stability and cap0-RNA–binding affinity, thereby promoting IFIT1 antiviral activity (13, 14).

Mouse models have been used extensively to examine the role of IFIT proteins in regulating human disease (15). Like human IFIT1, murine Ifit1 can bind to cap0 RNA with high affinity (16). *In vivo*, mutation of the virally encoded 2′-*O*-methyltransferase in a number of flavivirus and coronavirus species severely attenuated viral replication, and vaccination with these viruses can protect mice from challenge with virulent strains (17–22). Virulence was partially or fully restored upon Ifit1 knockout, indicating specific antiviral activity against cap0 viruses (17, 18, 21–23). However, recent phylogenetic analysis has concluded that murine Ifit1 and human IFIT1 are not orthologous, and these proteins have different antiviral activity against cap0 and cap1 viruses (4). Indeed, although human IFIT1 can bind to cap1-RNA with low affinity (8, 13), murine Ifit1 lacks any cap1-binding activity (4, 16).

Not only has IFIT1 been lost in mice and related mouse-like rodents, including model organisms such as the Norway rat and Chinese hamster, but IFIT5 is also absent (4). Instead, these species typically harbor multiple copies of the Ifit1b gene. In mice, Ifit1b has been duplicated twice, yielding three paralogues: Ifit1, Ifit1b, and Ifit1c (Fig. 1*A*) (also called Ifit1b1, Ifit1b2, and Ifit1b3, to reflect their evolutionary relatedness) (4). Despite the high degree of sequence identity between these paralogues, their functions remain unknown, and there is little evidence supporting their expression in mouse cells. In these species, Ifit3 has also undergone a 3′ truncation and lacks the potential to interact with murine Ifit1 (4, 13, 14). Therefore, rodent Ifits may have alternative mechanisms to regulate their expression and function, which are distinct from the human IFIT complex.

**Figure 1.**
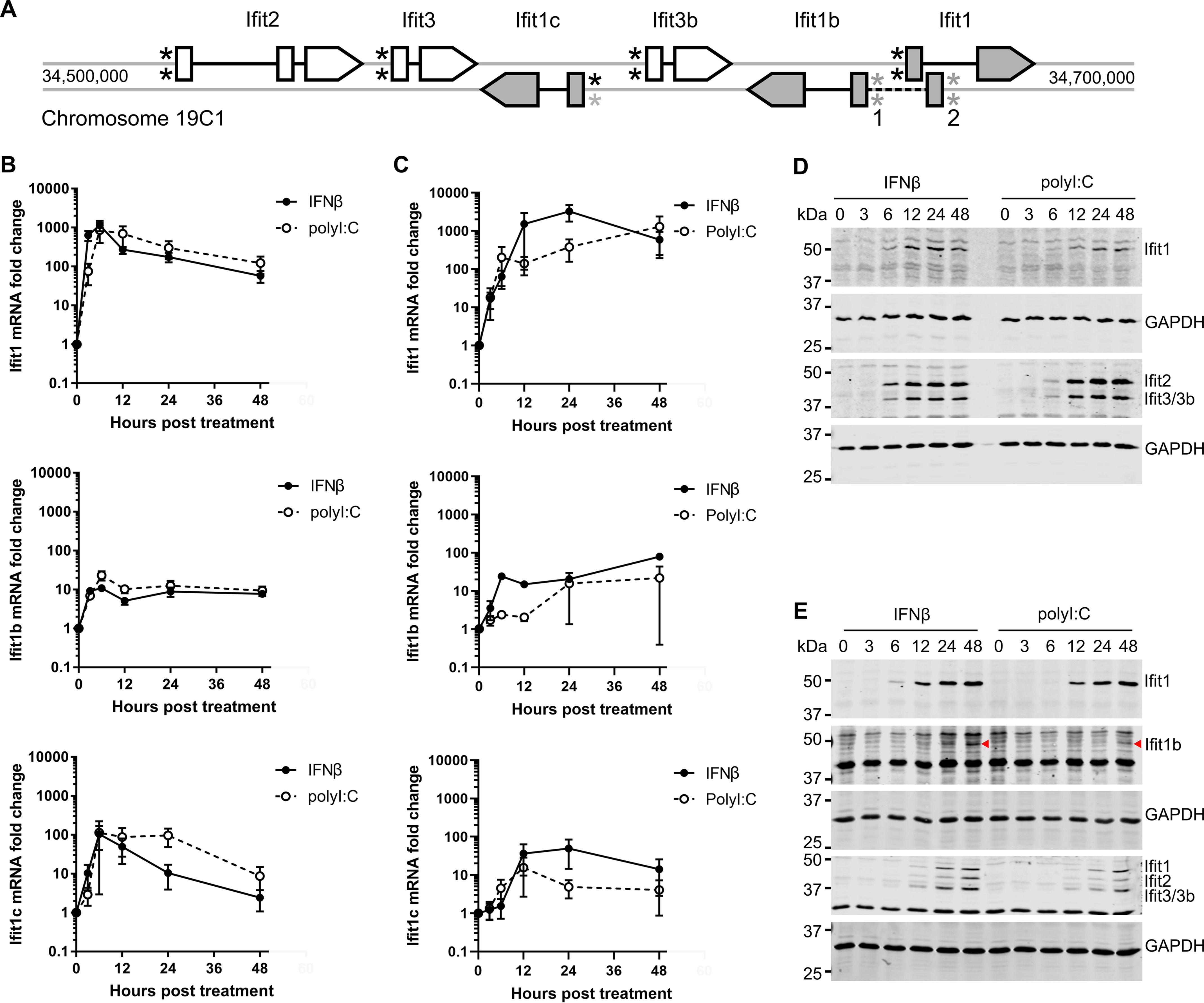
**Induction of Ifit gene expression in stimulated murine cells.** *A*, genome organization of the murine Ifit locus. Exons are shown as *boxes* with *arrows* indicating the direction of the reading frame, and introns as *solid black lines*. *Black asterisks* represent canonical ISREs, whereas *gray asterisks* indicate putative ISRE-like sequences. For Ifit1b, two transcription start sites are annotated. *B* and *C*, RT-qPCR analysis of RNA extracted from RAW264.7 cells (*B*) or 17Cl-1 cells (*C*) stimulated with IFNβ or transfected with poly(I:C) over 48 h. The graphs show the means and standard errors of two biological replicates. *D* and *E*, immunoblot analysis of RAW264.7 (*D*) or 17Cl-1 (*E*) cell lysates extracted at the same time as *B* and *C*. GAPDH is included as a loading control for each membrane. See also Fig. S1–S4.

In this study, we investigated the expression and activity of the entire murine Ifit family. We verified expression of noncanonical family members Ifit1b, Ifit1c, and Ifit3b in murine cells and found that Ifit1b binds to cap1-RNA with remarkable affinity and specificity. Ifit1b selectively inhibited cap1-RNA translation and restricted the replication of WT mouse coronavirus *in vitro*. We then established the different Ifit complexes that can form in mice and found that Ifit1c acts as a cofactor to Ifit1 and Ifit1b, promoting their stability and translation inhibition activity, thereby fulfilling a role analogous to human IFIT3. As such, this study helps to elucidate the ways in which primates and rodents have developed convergent roles for IFIT proteins in the innate immune response that occupy their same functional niche. We additionally highlight key distinctions between the human and murine IFIT families in the hope that this will inform use of mouse models in understanding IFIT biology and antiviral activity.

## Results

### Ifit1b, Ifit1c, and Ifit3b are expressed in murine cells following stimulation

To date, a systematic and quantitative examination of the induction kinetics of the entire murine Ifit family has not been carried out. Although the expression patterns of murine Ifit1, Ifit2, and Ifit3 have been examined in detail (24–27), the expression of the other three members of the murine Ifit family, Ifit1b, Ifit1c, and Ifit3b, has yet to be formally verified in mouse cells. To address this, murine Ifit expression was examined in RAW264.7 macrophage-like cells, 17Cl-1 immortalized fibroblasts, and murine embryonic fibroblasts (MEFs). The cells were stimulated with recombinant IFNβ or transfected with synthetic dsRNA (poly(I:C)) for up to 48 h. The cell lysates were examined by RT-qPCR and immunoblot analysis, using qPCR primers (Fig. S1) and antibodies (Fig. S2) specific to each murine Ifit family member. Because Ifit3 and Ifit3b differ by only 5 amino acids, it was not possible to differentiate between these proteins by immunoblotting so, to reflect this, this band will be annotated as Ifit3/3b.

**Figure 2.**
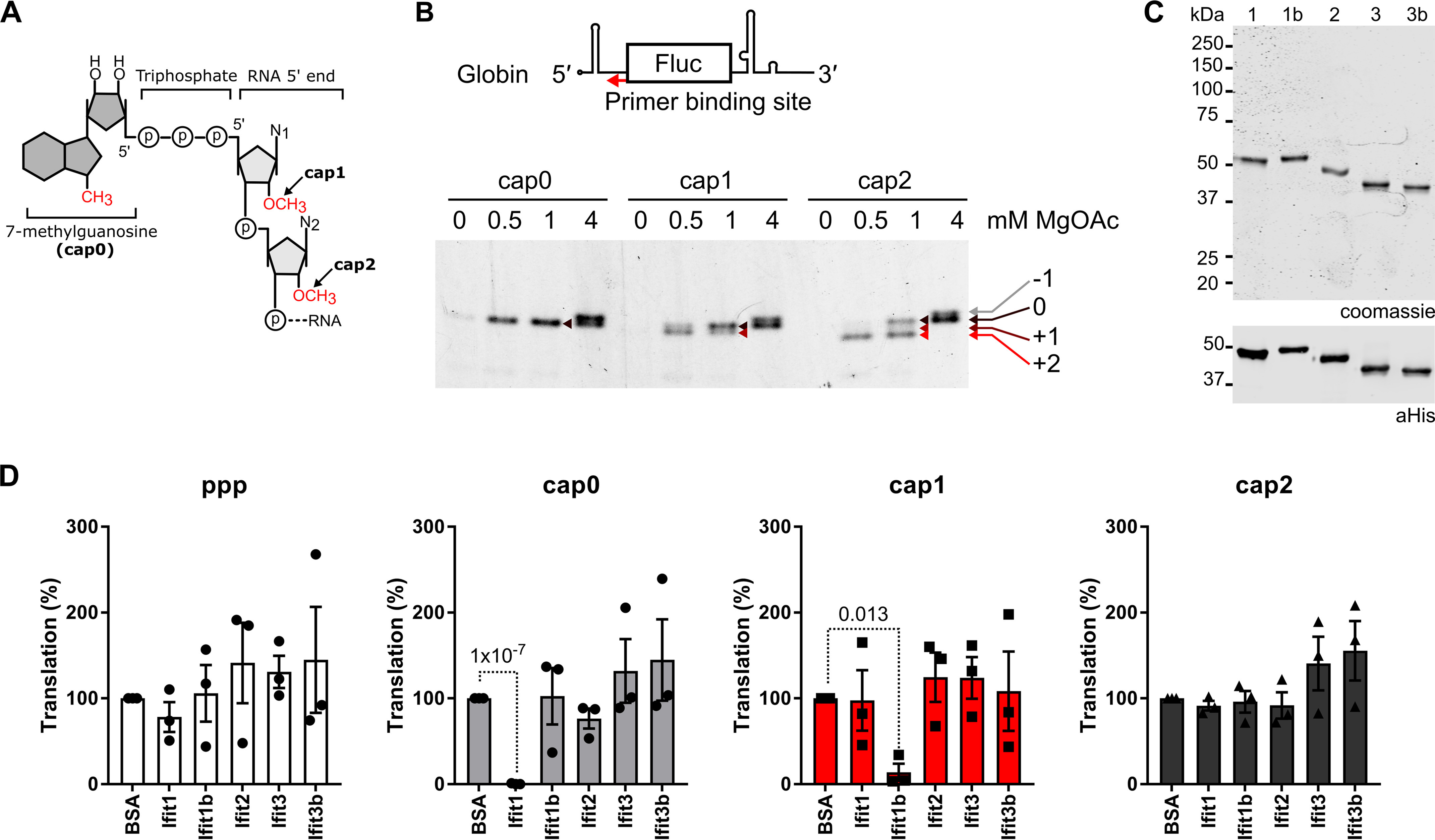
**Translation inhibition by murine Ifit proteins.** *A*, schematic of the mRNA 5′ cap. *B*, schematic of the Globin-Fluc mRNA, showing the primer-binding site for reverse transcription (*upper panel*) and primer extension analysis of capped Globin-Fluc mRNAs at different concentrations, to analyze cap methylation efficiency (*lower panel*). At lower Mg^2+^ concentrations, additional stops are visible corresponding to 2′-*O*-methylation of the first and second nucleotides, indicated with *arrowheads*. At high Mg^2+^ concentrations an additional band is seen at the −1 position, consistent with terminal transferase activity of the reverse transcriptase. *C*, SDS-PAGE (*upper panel*) and anti-His (*lower panel*) Western blotting of recombinant Ifit proteins. *D*, *in vitro* translation of differentially capped Globin-Fluc reporter mRNAs, normalized to the BSA-only control. The graphs show the means and the standard errors of at least three experiments. The data were compared with the BSA-only control by pairwise two-tailed *t* tests and *p* values < 0.1 are shown.

Consistent with previous reports (25, 26), Ifit1, Ifit2, and Ifit3 mRNA expression was rapidly induced following stimulation of RAW264.7 cells, with peak expression observed at 3–6 h poststimulation (Fig. 1*B* and Fig. S3*A*). Expression decreased between 9 and 24 h poststimulation. Ifit1, Ifit2, and Ifit3/3b proteins were detectable 6–12 h following stimulation, just after the peak of mRNA expression (Fig. 1*D*). Ifit mRNA expression was induced to a lesser extent in 17Cl-1 fibroblast cells, and expression was delayed compared with expression in RAW264.7 cells, with peak mRNA expression at 12–24 h poststimulation (Fig. 1*C* and Fig. S3*B*). Similarly, at the protein level, Ifit1, Ifit2, and Ifit3/3b were detectable at 24–48 h poststimulation (Fig. 1*E*), slightly later than in the RAW264.7 cells. In MEFs, the Ifit mRNA induction patterns were similar to RAW264.7 cells, although the magnitude of induction was 10–100-fold lower, and expression had largely returned to baseline by 24–48 h poststimulation (Fig. S3*C*). In both 17Cl-1 cells and MEFs, induction of Ifit2 expression was lower compared with RAW264.7 cells, indicating that Ifit2 may be regulated differently in fibroblasts compared with macrophages (Fig. 1, compare *D* and *E*, and Fig. S3).

**Figure 3.**
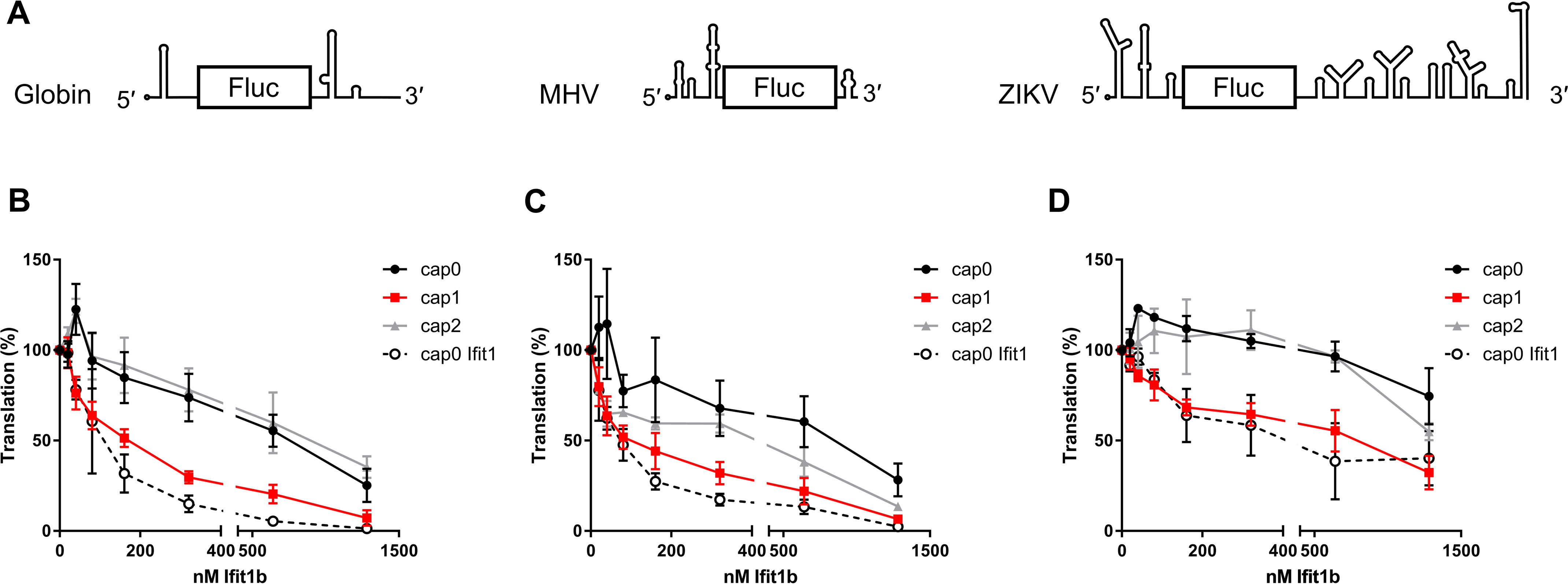
**Ifit1b inhibits translation of unstructured cap1-RNA.** *A*, schematics of reporter mRNAs used for *in vitro* translation. *B–D*, *in vitro* translation of differentially capped Globin-Fluc (*B*), MHV-Fluc (*C*), or ZIKV-Fluc (*D*), in RRL with increasing concentrations of Ifit1b, alongside titrations of Ifit1 on cap0 RNA for comparison (*dashed lines*). The data were normalized to the BSA-only control and shown as the means and the standard errors of at least two independent experiments. The IC_50_ values are listed in Table 1.

mRNA expression was observed for Ifit1b, Ifit1c (Fig. 1, *B* and *C*), and Ifit3b (Fig. S3) following stimulation and could be verified by Sanger sequencing of the qPCR product (Fig. S1). Although Ifit3b expression was strongly induced following stimulation, Ifit1b and Ifit1c were poorly up-regulated, with only 10–100-fold induction over baseline in all cell lines tested. We tested a number of antibodies to confirm Ifit expression at the protein level. A commercially available antibody against human IFIT1 was cross-reactive with murine Ifit1 but detected Ifit1b to a greater extent (Fig. S2*B*). This antibody could detect a signal in IFN-stimulated mouse cells, but because it reacted with both Ifit1 and Ifit1b, the identity of this signal was ambiguous. A peptide-raised antibody against Ifit1b detected Ifit1b without cross-reactivity with Ifit1 (Fig. S2*A*) with greater sensitivity (Fig. S2*C*). Using this antibody, in 17Cl-1 cell lysates, Ifit1b was detectable at the protein level 48 h (Fig. 1*E*) and 80 h (Fig. S2*D*) after IFN stimulation. A peptide-raised antibody against Ifit1c was highly specific for recombinant Ifit1c protein but could not reproducibly detect endogenous Ifit1c in stimulated mouse cells (Fig. S2*A*).

To investigate the reason behind the poor expression of Ifit1b and Ifit1c in murine cells, the promoter regions of Ifit1, Ifit1b, and Ifit1c were examined. Ifit1 has two well-defined tandem interferon-stimulated response elements (ISREs) within 100 bp of the transcription start site (TSS) (Fig. S4*A*). The Ifit1c promoter region contains one canonical ISRE sequence proximal to the TSS and a second ISRE-like sequence further upstream. For Ifit1b, there are two annotated TSS: one proximal to the coding sequence of Ifit1b (here designated Ifit1b_1) and one several kilobases upstream (Ifit1b_2), which overlaps the Ifit1 promoter region (Fig. 1*A*), both of which contain poorly conserved ISRE-like sequences (Fig. S4*A*).

**Figure 4.**
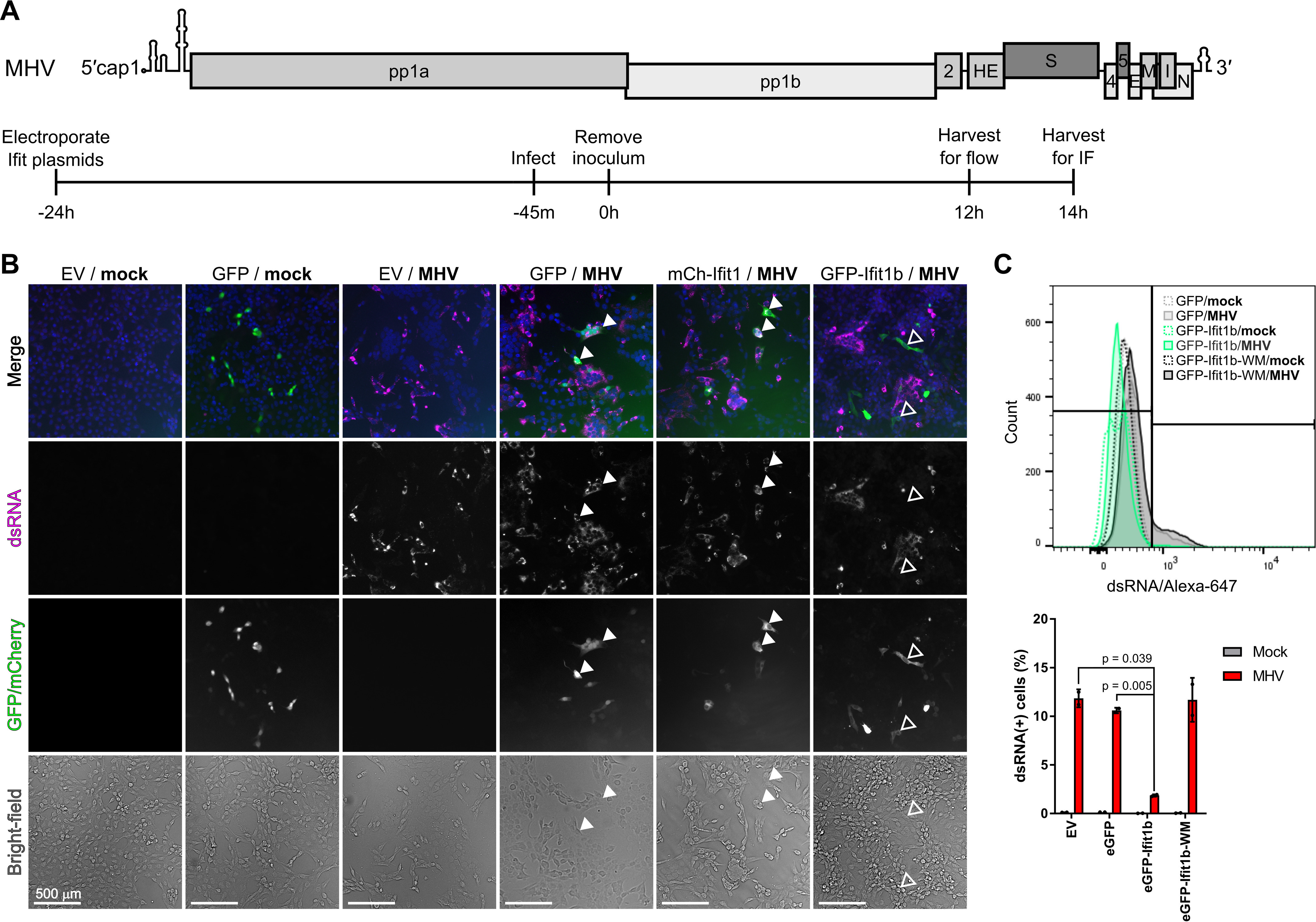
**Overexpression of Ifit1b inhibits MHV infection.** *A*, schematics of the MHV genome and experimental design. 17Cl-1 cells were electroporated with eGFP, eGFP-Ifit1b, mCherry-Ifit1, or empty vector (*EV*) 24 h before infection with MHV strain A59 at an MOI of 0.01 pfu/cell. 12 or 14 h after infection, the cells were fixed on coverslips for immunofluorescence microscopy or fixed in suspension for flow cytometry analysis, as indicated. The cells were stained for dsRNA as a marker for viral replication. *B*, immunofluorescence and bright-field microscopy of mock or MHV-infected cells. Fluorescently tagged Ifit proteins are shown in *green*, dsRNA is shown in *magenta*, and 4′,6-diamino-2-phenylindole–stained nuclei are shown in *blue*. *White arrowheads* highlight cells that are positive for GFP or mCherry-Ifit1, as well as dsRNA, whereas *open arrowheads* highlight cells expressing eGFP-Ifit1b that are dsRNA-negative. *C*, flow cytometry analysis of mock (*dashed lines*) or MHV-infected (*solid lines* with *shading*) cells transfected with GFP (*gray*), GFP-Ifit1b (*green*), or GFP-Ifit1b-WM, an RNA-binding mutant (*black*). The *black vertical line* indicates the gate for dsRNA-positive cells on the *x* axis. Quantification from this gate is shown in the *lower panel*. The data represent the means and standard deviations from two independent experiments. The data were compared by pairwise, two-tailed *t* tests assuming unequal variance, and *p* values <0.1 are shown. See also Fig. S5–S7.

Promoter plasmids were designed to express firefly luciferase (Fluc) under the control of the murine Ifit promoters. The promoter sequence was defined as 0.8–1 kb upstream of the TSS for each Ifit gene. A control plasmid was also generated with 1 kb of scrambled DNA sequence upstream of the Fluc mRNA (SCR). Promoter plasmids were cotransfected into 17Cl-1 cells alongside a constitutive *Renilla* luciferase (Rluc) expression plasmid. After 4 h, the cells were treated with IFNβ to stimulate promoter activity and then harvested after 24 h to determine luciferase expression. Luciferase activity was normalized to the Rluc control for each condition.

As expected, luciferase production from the Ifit1 promoter was strongly stimulated by treatment with IFNβ (Fig. S4*B*). For Ifit1b, the upstream Ifit1b_2 promoter weakly drove Fluc expression but was not IFN-responsive, whereas the downstream Ifit1b_1 promoter was slightly stimulated in response to IFNβ. Similarly, the Ifit1c promoter showed a small degree of up-regulation when cells were treated with IFNβ (Fig. S4*B*). Therefore, the lower expression of Ifit1b and Ifit1c at the mRNA level, described above, may be due to poorly IFN-responsive promoter sequences.

### Ifit1b specifically inhibits translation of cap1 mRNA

We next sought to examine the effect of the murine Ifit proteins on translation, using an *in vitro* translation assay system, which we have previously used to examine the effect of human IFIT heterocomplexing on their function (14). An Fluc reporter mRNA flanked by the 5′- and 3′-untranslated regions of human β-globin (Globin-Fluc) was transcribed and capped *in vitro* (Fig. 2, *A* and *B*). The efficiency of cap methylation was verified using a primer extension inhibition assay, based on the propensity of reverse transcriptase to terminate at methylated nucleotides, dependent on Mg^2+^ concentration (28, 29). Reverse transcription was carried out using avian myoblastosis virus (AMV) reverse transcriptase in the presence of a range of Mg^2+^ concentrations. At high Mg^2+^ concentrations, full-length signal was present for all mRNAs, as well as a proportion of 1 nt longer cDNAs, consistent with low-level terminal transferase activity (29). At lower Mg^2+^ concentrations, however, 1 or 2 nt shorter cDNA products were predominant for cap1 and cap2 RNA, respectively. At very low Mg^2+^ concentrations, only the lower band was detectable for cap1 and cap2 RNA, but not for cap0 RNA. This indicates high cap methylation efficiency, because no residual full-length signal was detectable (Fig. 2*B*).

Murine Ifit proteins were expressed and purified as described under “Experimental procedures,” with the exception of Ifit1c, which was poorly expressed and insoluble *in vitro*. Ifit proteins were normalized by Western blotting against the C-terminal His_8_ tag (Fig. 2*C*). Globin-Fluc reporter mRNAs were incubated with 500 nm recombinant Ifit protein before the addition of rabbit reticulocyte lysate (RRL), and translation was quantified by measuring luminescence from the Fluc reporter, normalized to the buffer-only control. Consistent with previous reports (4, 13, 16), Ifit1 strongly inhibited translation of the cap0 reporter but could not inhibit cap1 or cap2 translation (Fig. 2*D*). Translation from the uncapped reporter mRNA was much less efficient than any of the capped RNAs but was not reproducibly inhibited by any Ifit protein tested (Fig. 2*D*, *left panel*). Ifit2, Ifit3, and Ifit3b did not inhibit translation of any of the RNAs tested, consistent with the described activities of human IFIT2 and IFIT3 (5, 11, 12). However, Ifit1b strongly inhibited the translation of cap1 Globin-Fluc RNA but had no effect on cap0- or cap2-RNA translation (Fig. 2*D*).

### Ifit1b inhibits WT mouse coronavirus translation

To investigate Ifit1b cap1-RNA translation inhibition in more detail, titration experiments were performed using the Globin-Fluc reporter or reporters with viral 5′- and 3′-UTRs flanking the same Fluc ORF (Fig. 3*A*). Representative species of coronavirus (mouse hepatitis virus (MHV)) and flavivirus (ZIKV) were chosen, which have different degrees of RNA secondary structure at their 5′ ends (Fig. S5). Serial dilutions of Ifit1b were incubated with Fluc mRNAs bearing differentially methylated 5′ caps, and luciferase activity was used to monitor translation in RRL, as previously. 50% inhibitory concentrations were interpolated from the data and are presented in Table 1. Consistent with its described cap0-RNA–binding activity, Ifit1 caused a dose-dependent inhibition of cap0 globin-Fluc mRNA translation (IC_50_ = 102 nm; Fig. 3*B*, *dotted line*). Ifit1b inhibited cap1 Globin-Fluc translation at low concentrations, comparable with inhibition of cap0 mRNA by Ifit1 (IC_50_ = 152 nm; Fig. 3*B*, *red line*). However, even at the highest concentrations of Ifit1b tested, there was still a low level of cap1-RNA translation, compared with complete inhibition of cap0-RNA translation by Ifit1. Ifit1b only weakly inhibited cap0 and cap2 Globin-Fluc translation (IC_50_ = ∼675 and ∼825 nm, respectively).

**Figure 5.**
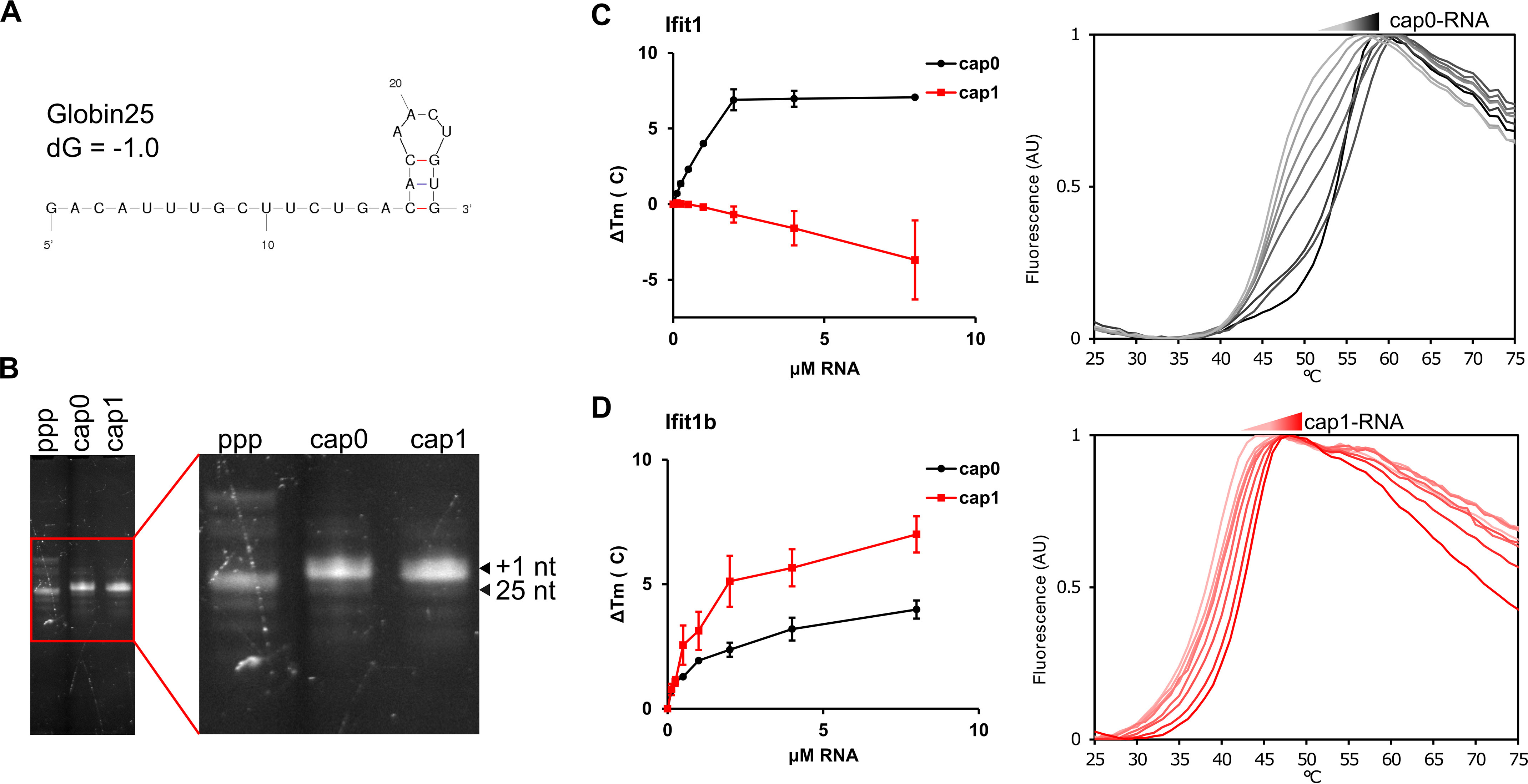
**RNA binding by Ifit1b.** *A*, secondary structure prediction of the first 25 nt of the β-globin 5′-UTR (globin25) RNA, calculated in Mfold (60). *B*, denaturing PAGE analysis of uncapped, cap0, and cap1 globin25 RNA. *C* and *D*, thermal stability analysis of Ifit1 or Ifit1b with increasing concentrations of cap0 (*gray*) or cap1 (*red*) globin25 RNA. Quantification is shown in the *left panels*, expressed as the increase in melting temperature (*T*_m_) over protein alone (*dT*_m_) (means ± S.E. from two-three independent experiments), and representative melt curves are shown in the *right panels*. *T*_m_ values were derived by nonlinear regression using the Boltzmann equation, *y* = *LL* (*UL* – *LL*)/(1 + exp(*T*_m_ – *x*)/*a*), where *LL* and *UL* are the lower and upper limits, respectively. See also Fig. S8.

**Table 1:** **Translation inhibition by murine Ifit proteins** The values are from the data presented in Fig. 3. The IC_50_ values are the concentrations of Ifit that reduce reporter translation by 50% ± standard error. The data were fitted to [Inhibitor] *versus* normalized response curve (*Y* = 100)/(1 + (*X*^HillSlope^)/(IC_50_^HillSlope^)) using the least-squares method in GraphPad Prism.

Ifit	RNA	IC_50_
		nm Ifit in RRL
Ifit1b	cap0-globin-Fluc	675 ± 129
Ifit1b	cap1-globin-Fluc	152 ± 18.1
Ifit1b	cap2-globin-Fluc	826 ± 183
Ifit1	cap0-globin-Fluc	102 ± 14.5
Ifit1b	cap0-MHV-Fluc	690 ± 233
Ifit1b	cap1-MHV-Fluc	101 ± 17.9
Ifit1b	cap2-MHV-Fluc	238 ± 50.1
Ifit1	cap0-MHV-Fluc	68 ± 9.5
Ifit1b	cap1-ZIKV-Fluc	1240 ± 300
Ifit1	cap0-ZIKV-Fluc	550 ± 240

MHV-Fluc mRNA was slightly more susceptible to translation inhibition by Ifit proteins. Ifit1 inhibited the translation of cap0-MHV-Fluc RNA at 1.6-fold lower concentrations compared with inhibition of cap0-Globin-Fluc (IC_50_ = 68 nm; Fig. 3*C*, *dotted line*). Likewise, Ifit1b inhibited the translation of cap1-MHV-Fluc RNA at 1.5-fold lower concentrations compared with cap1–Globin–Fluc RNA (IC_50_ = 101 nm; Fig. 3*C*, *red line*). Inhibition of the cap0-MHV reporter by Ifit1b was similar to the inhibition of the cap0-Globin reporter (IC_50_ ∼ 690 nm), supporting specificity for cap1-RNA binding over cap0. However, inhibition of cap2-MHV mRNA was slightly greater, indicating looser binding specificity to this reporter (IC_50_ = 238 nm). Therefore, the sequence or structure of MHV mRNA may alter cap-binding specificity, as well as affinity.

By contrast, ZIKV-Fluc mRNA translation was resistant to both Ifit1 and Ifit1b, even at micromolar concentrations (Fig. 3*D*). At the highest concentrations of Ifit1 or Ifit1b tested, ZIKV-Fluc translation was inhibited by a maximum of 30–50% by either Ifit1 or Ifit1b. We have previously shown that human IFIT1 could completely inhibit the translation of the same cap0 ZIKV-Fluc reporter at nanomolar concentrations and could inhibit cap1 ZIKV-Fluc translation at micromolar concentrations (14). Therefore, the inability of murine Ifit proteins to inhibit the translation of the same reporter mRNA is quite surprising.

Because Ifit1b could strongly inhibit the translation of cap1 MHV-Fluc reporter mRNAs, we reasoned that Ifit1b may be capable of inhibiting the lifecycle of (“restricting”) MHV in cell culture. To investigate this, plasmids encoding mCherry-tagged Ifit1, eGFP-tagged Ifit1b, or eGFP alone were electroporated into 17Cl-1 mouse fibroblast cells, which are permissive for MHV infection (30). After 24 h, the cells were infected with WT MHV strain A59 (genome structure shown in Fig. 4*A*, *upper panel*) at a multiplicity of infection (MOI) of 0.05 pfu/cell. After 16 h, the cells were harvested for immunofluorescence or flow cytometry analysis (experimental timeline shown in Fig. 4*A*, *lower panel*).

The cells on coverslips were fixed and stained for dsRNA, a well-described marker for RNA virus replication complexes (31). In empty vector or eGFP-transfected cells, 14 h after infection with MHV, the majority of cells showed dsRNA staining in the cytoplasm, whereas dsRNA was not visible in uninfected cells (Fig. 4*B*). eGFP-expressing cells were positive for dsRNA, indicating that overexpression of eGFP had no effect on viral replication. Similarly, many mCherry-Ifit1–expressing cells were also positive for dsRNA signal, indicating that Ifit1 is not directly antiviral (Fig. 4*B*, *white arrowheads*), although the dsRNA signal appeared to be lower in the cell population. By contrast, few cells overexpressing eGFP-Ifit1b were positive for dsRNA, implying a direct antiviral effect of Ifit1b on coronavirus infection (Fig. 4*B*, *open arrowheads*).

**Figure 6.**
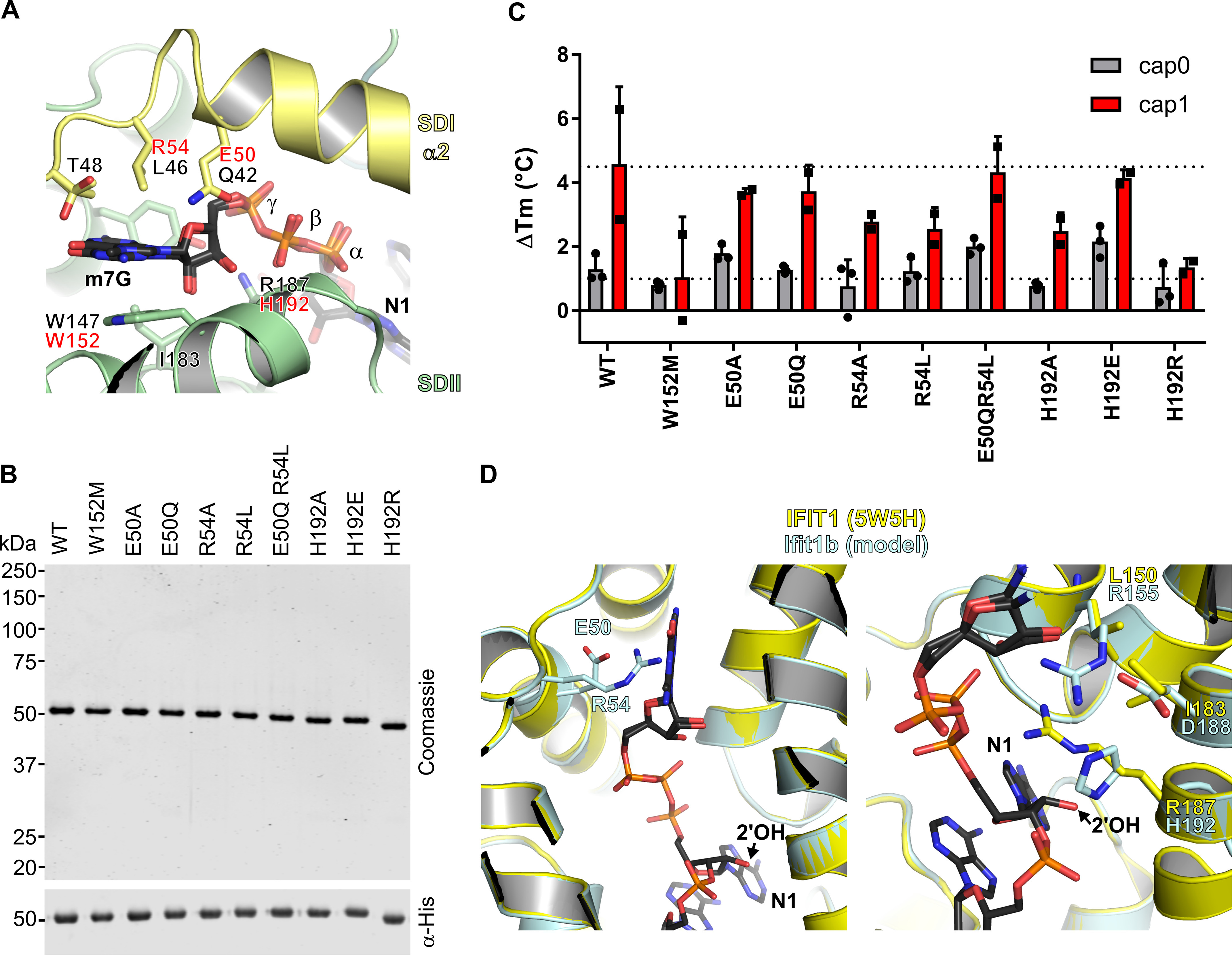
**Mutational analysis of Ifit1b RNA binding.** *A*, cartoon representation of the cap-binding pocket of human IFIT1 (Protein Data Bank entry 5W5H), colored by subdomain. Bound cap0 oligonucleotide A RNA is shown as *black* and *orange sticks*. Residues involved in human IFIT1 cap coordination and triphosphate binding are shown as *sticks* and labeled in *black*. The equivalent residues in murine Ifit1b, where divergent, are listed in *red*. *B*, Coomassie-stained SDS-PAGE (*upper panel*) and anti-His Western blotting (*lower panel*) of WT and mutant Ifit1b. *C*, thermal shift assay of 2 μm WT and mutant Ifit1b with 4 μm cap0 or cap1 RNA, showing the difference in melting temperature (*dT*_m_) between protein only and protein with RNA. The graph shows the means and standard deviations of three (cap0) or two (cap1) experimental replicates. *D*, a model of Ifit1b (*cyan*) was generated in SWISS-MODEL, based on the structure of human IFIT1 (Protein Data Bank entry 5W5H) and is shown superposed with human IFIT1 (*yellow*). *Left panel*, residues in the cap-binding loop, distal to the 2′-*O*-hydroxyl group of the first RNA nucleotide, that impact cap0/cap1 binding specificity are shown as *sticks*. *Right panel*, residues proximal to the first RNA nucleotide are shown. In murine Ifit1b, a number of mutations (relative to IFIT1) are present in this region, which may allow accommodation of 2′-*O*-methylated/cap1-RNA.

To quantify restriction by Ifit1b, 17Cl-1 cells were electroporated with Ifit expression plasmids, infected with MHV as previously, fixed in suspension, and stained for dsRNA, before analysis by flow cytometry (Fig. S6). Before infection, fluorescent protein expression was checked by microscopy at 20 h postelectroporation, and transfection efficiency was comparable between plasmids (Fig. S6*A*). The eGFP-Ifit1b–transfected cell population had much lower dsRNA signal compared with empty vector or eGFP-transfected cells, indicating that it was resistant to infection with MHV (Fig. 4*C*). Importantly, cells transfected with a mutant of Ifit1b that does not bind to cap1-RNA (Ifit1b-WM; described further in Fig. 6) were infected to the same extent as eGFP- or empty vector–transfected cells, indicating that Ifit1b restricts MHV infection in a manner dependent on its RNA-binding activity. eGFP-Ifit1b and eGFP-Ifit1b-WM expression was equivalent when analyzed by flow cytometry (Fig. S6*C*). Therefore, these data support an antiviral role for Ifit1b in MHV infection and correlates with an inhibition of viral translation by binding to the cap1-RNA genome.

By contrast, infection of mCherry-Ifit1-transfected cells was similar to infection of empty vector–transfected cells, indicating that Ifit1 does not inhibit MHV infection in these cells (Fig. S6*E*). This is consistent with the inability of Ifit1 to bind to cap1-RNA. However, we have previously observed that murine Ifit1 may promote type I IFN expression in mouse cells and thereby restrict mouse norovirus infection (32). Because 17Cl-1 cells respond slowly to dsRNA (Fig. 1) and do not up-regulate ISG expression during acute MHV infection (33), this may explain why we did not observe an antiviral phenotype for Ifit1 in this cell type.

### Ifit1b regulates host translation

It has been estimated that between 30 and 70% of mouse mRNA transcripts have cap1 5′ ends, with the remainder bearing cap2 (34), whereas cap0 5′ ends are undetectable in various human and mouse cells (35). Therefore, we hypothesized that Ifit1b should be capable of inhibiting the translation of a proportion of host mRNA transcripts. To investigate this, a puromycylation-labeling approach was taken (36). Puromycin is an antibiotic that mimics the structure of aminoacylated tRNA and is thus incorporated into the nascent polypeptide chain during elongation, resulting in premature chain termination. When mammalian cells are treated with low concentrations of puromycin, it is stochastically incorporated toward the C termini of nascent polypeptides. Using antibodies raised against puromycin, these labeled proteins can be detected by Western blotting, thereby allowing visualization of the nascent proteome of the treated cell.

Murine 17Cl-1 fibroblast cells were transfected with FLAG-tagged Ifit1 or Ifit1b for 16 h before treatment with 5 µg/ml puromycin for a further 4 h. The cell lysates were separated by SDS-PAGE and transferred to nitrocellulose membranes, which were stained with REVERT total protein stain to ensure an equal quantity of lysate was loaded in each well (Fig. S7). The membranes were then analyzed by immunoblotting, using a mAb against puromycin. Cells overexpressing Ifit1b showed a 30% reduction in puromycin incorporation, compared with the empty vector control, indicating that Ifit1b can indeed inhibit a proportion of cellular translation (Fig. S7). Cells overexpressing Ifit1 showed similar levels of incorporation to empty vector–transfected cells, indicating little effect on cellular translation.

**Figure 7.**
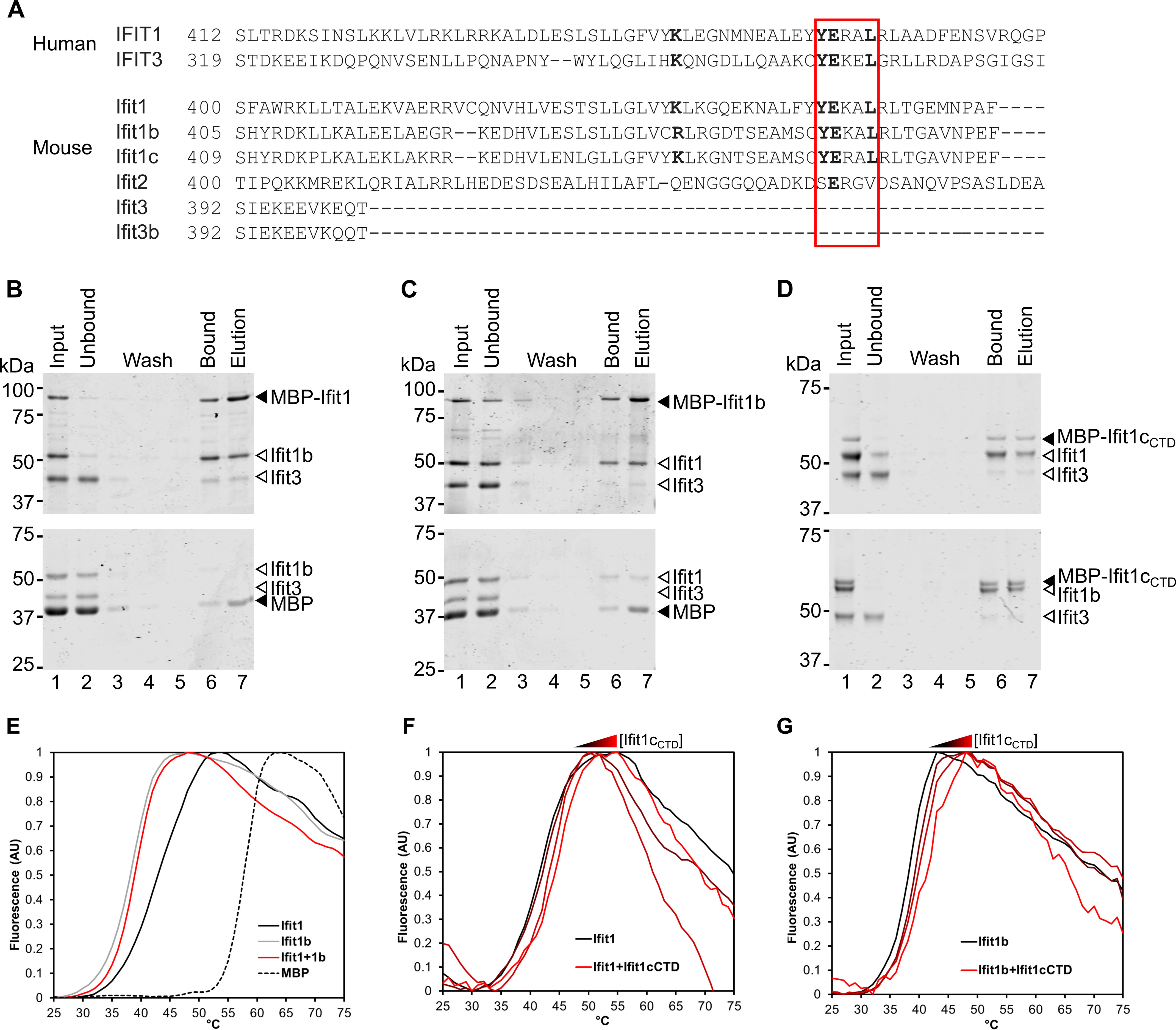
**Murine Ifit heterocomplex formation.** *A*, sequence alignment, showing the conserved Y*XXX*L interaction motif (*red box*), which mediates interaction between human IFIT1 and IFIT3 (*uppercase labels*), and its conservation in murine Ifit proteins (*lowercase labels*). *B–D*, coprecipitation of Ifit1, Ifit1b, and truncated Ifit1c (Ifit1c_CTD_). MBP-tagged bait, or MBP alone, was incubated with prey proteins (*lane 1*) before binding to amylose resin. Unbound proteins were washed away (*lanes 2–5*), and bound proteins remained on the beads (*lane 6*). The bound proteins were eluted in maltose-containing buffer (*lane 7*). Ifit3 was included as a negative control in each experiment. *E–G*, thermal stability analysis of Ifit proteins and complexes. In *F* and *G*, *brighter shades of red* indicate higher concentrations of Ifit1c_CTD_. See also Fig. S9–S12 and Table 2.

### Ifit1b binds specifically to cap1 RNA

Ifit1b has previously been hypothesized not to bind to RNA because it has residues in the cap-binding pocket and RNA-binding channel that could disrupt association with RNA, based on structural and mutational analysis of human IFIT1 (8). Therefore, we wanted to determine whether inhibition of cap1 translation by Ifit1b was due to RNA binding or a different mode of action. Previously, we have used a primer extension inhibition approach to determine the RNA-binding affinity of IFIT proteins. However, unlike Ifit1, Ifit1b-RNA binding could not be visualized by primer extension (Fig. S8).

**Figure 8.**
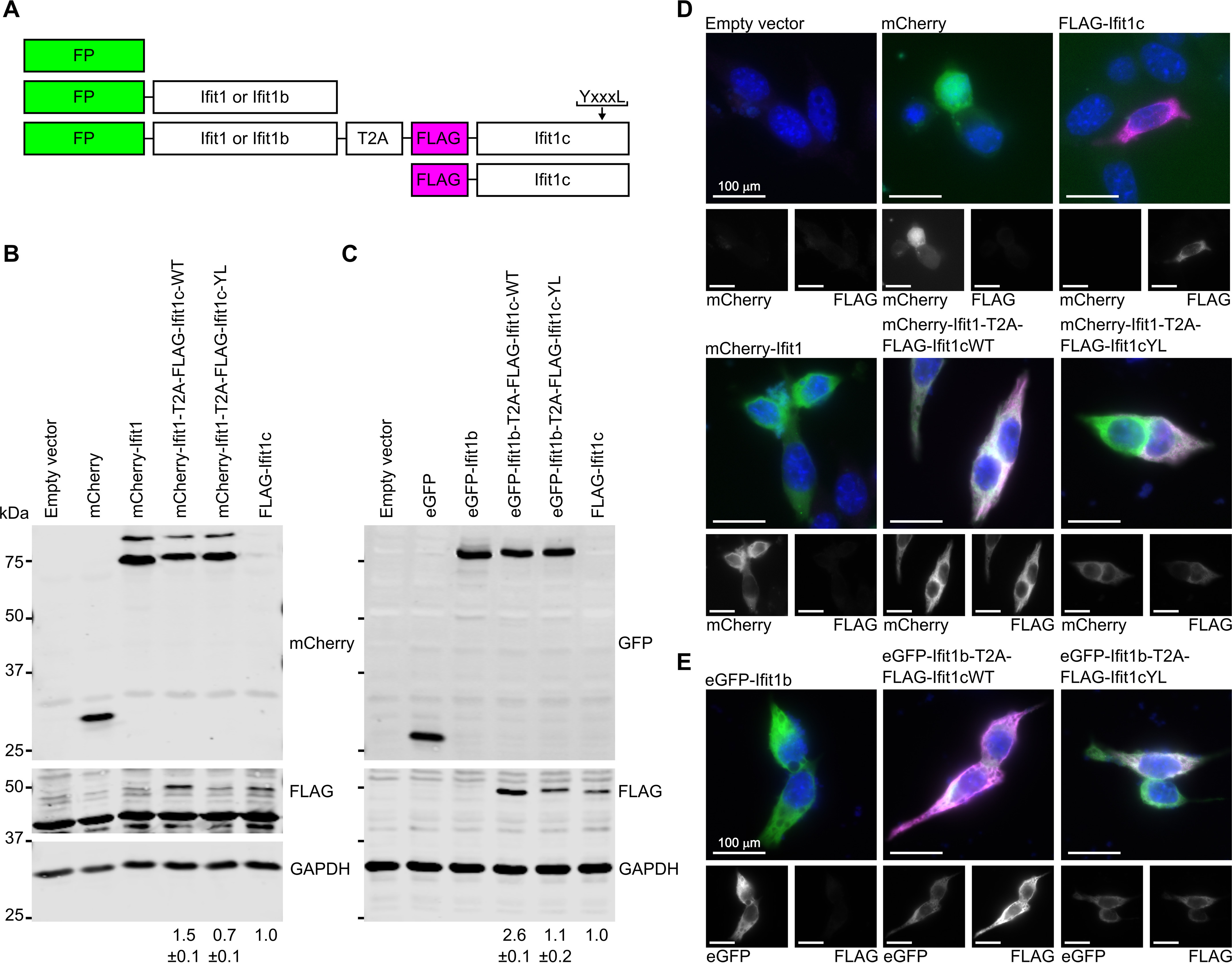
**Heterocomplexing enhances murine Ifit stability in mouse cells.** *A*, schematics of Ifit coexpression plasmids. *FP*, fluorescent protein (mCherry or eGFP); *T2A*, thosea asigna virus 2A Stop-Go sequence. The Ifit1c sequence was either WT or contained mutations in the C-terminal domain to disrupt binding to Ifit1 or Ifit1b (Y456E/L460E, *YL*). *B–E*, 17Cl-1 cells were transfected with control or Ifit expression plasmids for 24 h and then harvested for immunoblotting (*B* and *C*) or stained for immunofluorescence (*D* and *E*). In *B* and *C*, quantification of FLAG signal, normalized to GAPDH, is shown *below* each lane. The data represent the means and standard deviations of two biological repeats. For consistency and visual clarity in *D* and *E*, the micrographs have been pseudocolored such that fluorescent proteins (both mCherry and eGFP) are shown in *green*, and anti-FLAG signal is shown in *magenta*. The micrographs are representative of at least two independent experiments. See also Fig. S13.

As an alternative approach, a thermal stability assay was developed to examine Ifit1b-RNA binding. This technique employs a dye that fluoresces when it binds to hydrophobic patches exposed as a protein unfolds to quantify protein melting temperature (37). Binding to a substrate can increase the thermal stability of proteins, resulting in an increase in a melting temperature that correlates with its binding kinetics (38). Because it is known that IFIT proteins adopt more stable “closed” conformations upon RNA binding (7), we reasoned that RNA binding should stabilize Ifit melting temperature.

Ifit proteins were melted in the presence of increasing concentrations of an RNA oligonucleotide, comprising the first 25 nucleotides of the human β-globin 5′-UTR (globin25), which is predicted to be unstructured (Fig. 5*A*). A short oligonucleotide was chosen to minimize nonspecific stabilization, for example resulting from interactions between the body of the RNA and the surface of the Ifit protein. Globin25 oligonucleotides were purified by size-exclusion chromatography to remove small molecule contaminants, and capping efficiency was confirmed by high resolution denaturing PAGE (Fig. 5*B*). Because both specific and nonspecific binding can contribute to protein stabilization, heterologous yeast tRNA was included as a blocking agent, such that only specific, high-affinity interactions that are sufficient to displace bound tRNA produce a signal above baseline.

Increasing concentrations of cap0 RNA resulted in a dose-dependent stabilization of Ifit1, as expected (Fig. 5*C*). Cap1 RNA did not stabilize Ifit1 but actually slightly reduced Ifit1 melting temperature (Fig. 5*C*). Consistent with the translation inhibition assays, Ifit1b was stabilized in a dose-dependent manner by cap1-globin25 RNA, indicative of binding (Fig. 5*D*). Stabilization by cap0-globin25 RNA was lower, supporting specific binding to cap1-RNA over cap0-RNA.

To investigate the mechanism of RNA binding by Ifit1b, we generated a panel of point mutants based on homology modeling (see “Experimental procedures”) with the human IFIT1/cap0 RNA structure (Fig. 6*A*). The mutant proteins were purified and normalized by immunoblotting against the C-terminal His_8_ tag (Fig. 6*B*). They were then incubated with a 2-fold molar excess of cap0- or cap1-globin25 RNA, before thermal stability analysis. Mutation of a conserved tryptophan residue, Trp^152^, which is necessary for cap guanosine coordination by human IFIT1 (8), reduced stabilization of Ifit1b by cap1-RNA back to background levels (Fig. 6*C*). Mutation of the charged residues in the cap-binding loop had little effect on cap1-RNA binding. Mutation of Glu^50^ to alanine or glutamine only slightly reduced cap1 RNA binding, whereas mutation of Arg^54^ to alanine or leucine reduced stabilization by cap1 RNA by about half. Mutation of both Glu^50^ and Arg^54^ to the equivalent residues in Ifit1 and conserved in human IFIT1 (glutamine and leucine, respectively) restored cap1 binding back to WT levels (Fig. 6*C*). Previously, in human IFIT1, mutation of these residues to alanine only slightly reduced cap0-RNA binding (8), indicating that they contribute to stable cap binding, but their exact identity is not critical. We also observed a slight increase in cap0-RNA binding by the E50A and E50Q/R54L mutants, indicating that they may impact RNA-binding specificity, even though these residues do not contact the first RNA nucleotide (Fig. 6*D*, *left panel*). Together, these results indicate that Ifit1b likely engages the cap using the conserved tryptophan 144 residue, but the other face of the cap-guanosine is coordinated nonspecifically, in this case by long, polar side chains in the cap-binding loop, which may impact cap-binding specificity.

Next, mutations were made within the RNA-binding channel to investigate how Ifit1b achieves specific cap1-RNA binding. In human IFIT1, the residues immediately proximate to the ribose 2′-hydroxyl group, Tyr^157^ and Arg^187^, sterically hinder binding to 2′-*O*-methylated RNA (8) (Fig. 6*D*, *right panel*). In murine Ifit1b, the tyrosine is conserved at position 162, but the arginine residue is substituted for His^192^. Therefore, His^192^ was investigated for its contribution to RNA methylation sensing, by mutation to alanine, arginine, or glutamate. Mutation to alanine reduced stabilization by cap1 RNA by half, whereas mutation to glutamate had no effect on cap1-RNA binding. However, H192E increased stabilization of Ifit1b by cap0 RNA by 2-fold, indicating that His^192^ may indeed play a role in discriminating RNA methylation state (Fig. 6*C*). Mutation of His^192^ to arginine, mimicking human IFIT1 and murine Ifit1, abrogated RNA binding entirely. However, Ifit1b H192R was less stable than WT Ifit1b, indicating that H192R may disrupt the correct folding of Ifit1b, accounting for the loss of RNA-binding activity. The reciprocal in human IFIT1 (R187H) similarly abolished cap0-RNA binding (8).

### Murine Ifit proteins form heterodimeric complexes

We and others recently reported that the interaction between human IFIT1 and IFIT3 is important for regulation of IFIT1 activity (13–15). However, murine Ifit3 cannot interact with murine Ifit1 (13, 16), because of a genetic truncation in mouse-like rodents that deletes the region of the protein responsible for IFIT1 interaction (Fig. S9). However, the C-terminal Y*XXX*L motif, critical for human IFIT1-IFIT3 complex formation, is conserved in murine Ifit1, Ifit1b, and Ifit1c, indicating that these proteins may be capable of heterocomplexing (Fig. 7*A* and Fig. S10).

**Figure 9.**
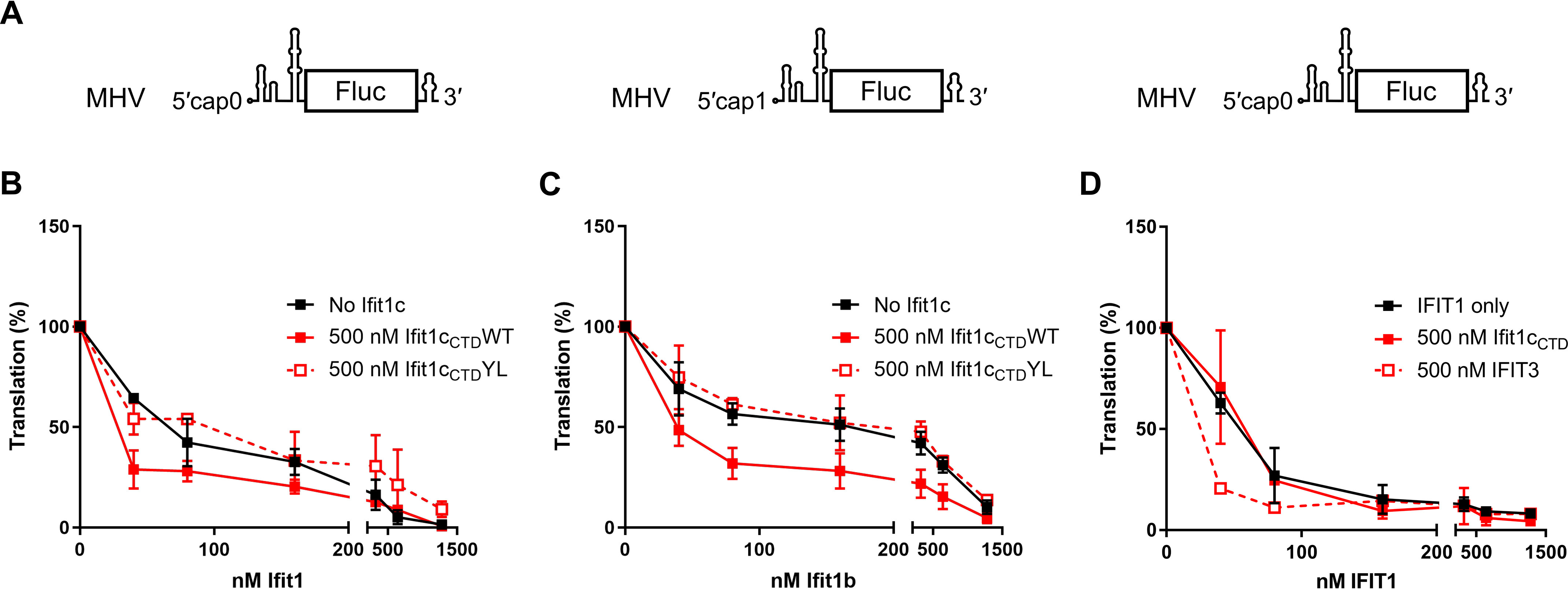
**Ifit1c enhances translation inhibition by Ifit1 and Ifit1b.** *A*, schematics of reporter mRNAs used for *in vitro* translation. *B–D*, *in vitro* translation of MHV-Fluc reporter mRNAs in RRL with increasing concentrations of Ifit1 (*B*), Ifit1b (*C*), or human IFIT1 (*D*), in the presence or absence of 500 nm Ifit1c_CTD_, either WT or Y*XXX*L mutant (*YL*). The data were normalized to luciferase activity in the absence of IFIT protein and shown as the means and standard errors of at least two independent experiments. See Table 3 for calculated IC_50_ values.

**Figure 10.**
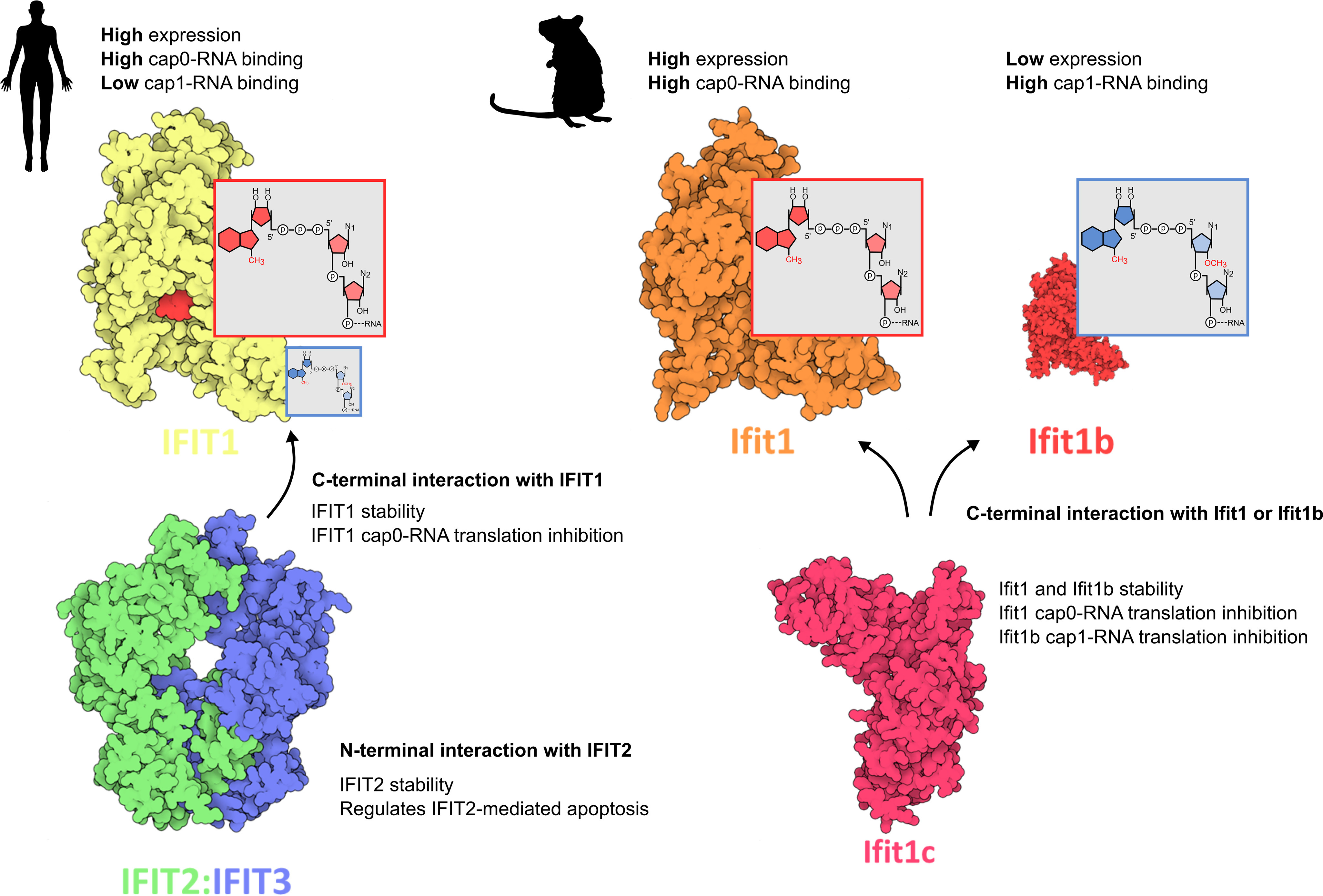
**Model for human IFIT and mouse Ifit function.** During an antiviral response in humans, IFIT1 is highly expressed following IFN stimulation. Human IFIT1 binds strongly to cap0-RNA (*red*) but weakly to cap1-RNA (*blue*) and inhibits their translation. Cap0-RNA is associated with viral infection and is recognized as “nonself,” whereas cellular RNA is typically cap1 modified and is therefore recognized as “self.” As such, IFIT1 strongly inhibits the translation of nonself viral RNA while only weakly inhibiting self cellular RNA. In stimulated mouse cells, murine Ifit1 is strongly expressed and binds to cap0-RNA, whereas murine Ifit1b is poorly expressed and binds strongly to cap1-RNA. In this way, murine Ifit1 and Ifit1b acting together can strongly inhibit the translation of nonself viral RNA while weakly inhibiting self cellular RNA. Human IFIT1 stability and activity is regulated by human IFIT3, which interacts via a conserved C-terminal interaction motif. Human IFIT3 also interacts with human IFIT2, via the N terminus, and regulates IFIT2-mediated apoptosis. As such the human IFIT1-IFIT2-IFIT3 complex is coregulated and is involved in multiple stages of the antiviral response. Murine Ifit1 and Ifit1b are both regulated by murine Ifit1c, which also interacts via the C-terminal domain. Ifit1, Ifit1b, and Ifit1c do not bind to Ifit3; therefore the functions of Ifit heterocomplexes in mice are regulated separately.

We first examined the oligomeric state of Ifit proteins in solution. Human IFIT1 was recently described to homodimerize in a concentration-dependent manner, via the C-terminal Y*XXX*L motif (39). BSA, which is a 65-kDa monomer with a small proportion of 132-kDa dimeric species, was used as a size marker for size-exclusion chromatography (SEC). When analyzed by SEC, Ifit1 and Ifit1b eluted after the BSA dimer but before the BSA monomer peak, indicating that these proteins are homodimers (Fig. S11, *A* and *B*). This was confirmed for Ifit1b, which had the molecular weight of a dimer when analyzed by SEC coupled with multiangle light scattering (SEC-MALS) at different concentrations (Fig. S11*C*). When the Y*XXX*L motif was mutated in either Ifit1 or Ifit1b, the mutant proteins eluted later on the SEC column, after the BSA monomer, indicating that they are indeed monomeric (Fig. S11, *A* and *B*). When analyzed by SEC, MBP-Ifit1c_CTD_ eluted in the void fraction, consistent with the poor stability of this protein and its tendency to aggregate, and so was not suitable for SEC-MALS analysis.

To investigate which murine Ifit proteins can interact with each other, an *in vitro* coprecipitation assay was used, similar to that employed by Johnson *et al.* (13) for the interrogation of human IFIT interactions. Equimolar MBP-tagged bait and His-tagged prey proteins were incubated together at 30˚C for 1 h, before precipitation on amylose resin. Ifit3 was included as a negative control in each experiment and, consistent with a recent report (13), did not coprecipitate with any of the baits tested (Fig. 7, *B–D*). MBP-Ifit1 precipitated both Ifit1b and Ifit1 (Fig. 7*B* and S12*A*), whereas MBP-Ifit1b precipitated both Ifit1 and Ifit1b (Fig. 7*C* and Fig. S12*B*), indicating that Ifit1 and Ifit1b are capable of heterocomplexing. When Ifit1 and Ifit1b were incubated together and analyzed by SEC, the eluting species was the same size as the Ifit1 or Ifit1b homodimers, and higher order species were not detected (Fig. S11*D*). MBP alone did not interact with Ifit1b or Ifit3 (Fig. 7*B*, *lower panel*) but pulled down a trace amount of Ifit1 (Fig. 7*C*, *lower panel*). Because Ifit1 had a tendency to precipitate during this assay, this likely represents nonspecific binding of Ifit1 to the MBP or to the beads themselves.

To determine whether Ifit1 or Ifit1b could interact with Ifit1c, full-length MBP-tagged Ifit1c was used as bait. Recombinant full-length Ifit1c was highly unstable and, despite exhaustive efforts, refractory to purification. MBP-tagged Ifit1c was soluble but was nevertheless highly impure (Fig. S12, *C* and *D*). However, despite the contaminants present in the recombinant MBP-Ifit1c, Ifit1b was clearly visible in the precipitate, indicative of an interaction (Fig. S12*D*). Because of the presence of a contaminant band in Ifit1c at the same molecular weight as Ifit1, this interaction was more difficult to confirm (Fig. S12*C*). To circumvent this, a truncated MBP-tagged Ifit1c construct was generated, containing the three most C-terminal tetratricopeptide repeats (MBP-Ifit1c_CTD_, amino acids 338–470; Fig. S12*E*), which allowed purification of clean recombinant Ifit1c for use as bait. Ifit1 and Ifit1b both precipitated with MBP-Ifit1c_CTD_, whereas Ifit3 did not (Fig. 7*D*). Therefore, Ifit1c can specifically form complexes with both Ifit1 and Ifit1b via its C-terminal domain.

The relative affinity of these interactions was investigated by competitive coprecipitation experiments. MBP-tagged Ifits were used as bait and incubated with prey protein at 30 ˚C before binding to amylose resin, as previously. The beads were then washed with increasing concentrations of a competitor prey protein, with the expectation that higher affinity interactions should displace lower affinity ones. However, we observed that even high concentrations of Ifit1b could not disrupt the interaction between Ifit1 and MBP-Ifit1c_CTD_ (Fig. S12*F*), and likewise Ifit1 did not disrupt the Ifit1b-MBP-Ifit1c_CTD_ complex (Fig. S12*G*). Next, MBP-Ifit1c_CTD_ was incubated together with both Ifit1 and Ifit1b, at different temperatures. When incubated together on ice MBP-Ifit1c_CTD_ coprecipitated both Ifit1 and Ifit1b, to a similar extent (Fig. S12*H*). Because this interaction occurred even at low temperatures, it indicates that heterocomplexing is preferential and high affinity, but Ifit1c binds to both Ifit1 and Ifit1b with comparable affinity. Slightly more Ifit1 coprecipitated with MBP-Ifit1c_CTD_ when the proteins were incubated at 30 ˚C (Fig. S12*I*), which may be indicative of aggregation, rather than true preferential interaction, as we observed with MBP-only (Fig. 7*C*, *lower panel*). Therefore, these experiments indicate that purified Ifit1c can interact with both Ifit1 and Ifit1b to a similar extent, and these interactions are preferential over homodimerization.

### Heterocomplexing enhances Ifit stability in vitro

We previously observed that functionally important human IFIT heterocomplexes were more stable than IFIT proteins in isolation, both *in vitro* and in human cells (14). Therefore, we analyzed the thermal stability of murine Ifit proteins and complexes using differential scanning fluorimetry. Ifit proteins were incubated alone or in combination for 30 min on ice and then assayed for thermal stability, as described above for the Ifit1b RNA-binding assay. Full-length Ifit1c could not be used because of the high degree of contaminants present, so instead the C-terminal fragment of Ifit1c was analyzed. Ifit1c_CTD_ was expressed without an MBP tag, because MBP is very stable and would interfere with the melt curve analysis.

Murine Ifit1 and Ifit1b alone were relatively unstable, with melting temperatures around physiological temperature (Fig. 7*E* and Table 2). For comparison, MBP had a melting temperature of 60 °C when analyzed under the same conditions (Fig. 7*E*, *dashed line*). When Ifit1 and Ifit1b were mixed together, the melt curve was intermediate between the melting temperatures of the constituent proteins, indicating that this interaction does not provide any stability to either protein (Fig. 7*E*). Ifit1c_CTD_ alone was very unstable and did not produce a quantifiable melt curve. However, when either Ifit1 or Ifit1b was mixed with increasing concentrations of Ifit1c_CTD_, melting temperature increased by up to 3 ˚C (Fig. 7, *F* and *G*, and Table 2). Therefore, interaction with the C-terminal domain of Ifit1c enhances the thermal stability of both Ifit1 and Ifit1b.

**Table 2:** **Melting temperatures of Ifit proteins and complexes** Melting temperatures (*T*_m_) were interpolated from the data presented in Fig. 6. The data were analyzed by nonlinear regression using the Boltzmann equation: *y* = *LL* (*UL* – *LL*)/(1 + exp(*T*_m_ – *x*)/*a*), where *LL* and *UL* are the lower and upper limit, respectively.

Protein or complex	*T*_m_
	°*C*
Ifit1	41.9
Ifit1b	38.3
Ifit1 + Ifit1c_CTD_	44.4
Ifit1b + Ifit1c_CTD_	41.0
MBP	57.7

### Ifit1 and Ifit1b stabilize Ifit1c expression in murine cells

Ifit heterocomplexes were then examined in mouse cells, to determine whether coexpression of different murine Ifit proteins can stabilize their expression. To do this, plasmids were generated that expressed mCherry-tagged Ifit1 or eGFP-tagged Ifit1b, followed by FLAG-tagged Ifit1c in the same ORF, separated by the 2A stop-go peptide sequence from thosea asigna virus. The 2A sequence efficiently skips a peptide bond during translation elongation, effectively cleaving the two proteins (40), allowing stoichiometric coexpression of Ifit proteins from the same plasmid. Ifit1c was either WT or had a mutant Y*XXX*L (YL) motif to disrupt heterocomplexing. For consistency and clarity, in Fig. 8 both mCherry-Ifit1 and eGFP-Ifit1b fluorescent proteins are shown in *green*, whereas FLAG-Ifit1c is shown in *magenta* (Fig. 8*A*).

Murine 17Cl-1 cells were seeded onto coverslips and then transfected with Ifit coexpression plasmids. After 24 h, the cells on coverslips were fixed and stained for FLAG, whereas surrounding cells from the same well were harvested for Western blotting. When transfected alone, FLAG-Ifit1c expression was very low (Fig. 8, *B* and *C*). However, FLAG-Ifit1c expression was moderately enhanced when coexpressed with mCherry-Ifit1 (Fig. 8*B*) and strongly enhanced by coexpression with eGFP-Ifit1b (Fig. 8*C*). However, when the Y*XXX*L motif in Ifit1c was mutated, expression was similar to Ifit1c alone (Fig. 8, *B* and *C*), indicating that interaction with Ifit1 or Ifit1b is necessary for Ifit1c stabilization. Neither mCherry-Ifit1 nor eGFP-Ifit1b expression was affected by coexpression with WT or mutant Ifit1c (Fig. 8, *B* and *C*).

When analyzed by microscopy, mCherry-Ifit1 or eGFP-Ifit1b expressed alone showed diffuse cytoplasmic localization (Fig. 8, *D* and *E*, *left-most panels*), typical of IFIT proteins (41, 42). However, when FLAG-Ifit1c was expressed alone, it showed punctate staining within the cytoplasm, with few cells showing diffuse localization (Fig. 8*D*, *top right panel*). These puncta did not significantly colocalize with a proteasome marker and did not aggregate at the proteasome when cells were treated with the proteasome inhibitor MG-132 (Fig. S13*A*). FLAG-Ifit1c expression was also not rescued upon MG-132 treatment (Fig. S13*B*).

When WT FLAG-Ifit1c was coexpressed with either mCherry-Ifit1 or eGFP-Ifit1b, both proteins localized in the cytoplasm, and very few cells showed punctate staining for Ifit1c (Fig. 8, *D*, *center bottom panels*, and *E*, *center panel*). Ifit1c expression also appeared to be higher, evident from the brighter fluorescence signal, consistent with the Western blotting data (Fig. 7*D*, compare *top right panel* with *center bottom panels*). When FLAG-Ifit1c-YL was coexpressed with mCherry-Ifit1 or eGFP-Ifit1b, the cells still expressed both signals in the cytoplasm. However, colocalization of the two signals was not as consistent as for WT Ifit1c, and many cells still showed punctate FLAG staining (Fig. 7, *D*, *right bottom panels*, and *E*, *right panel*). The fluorescence signal was also weaker for FLAG in these cells, indicating lower Ifit1c expression. Therefore, interaction between Ifit1 or Ifit1b and Ifit1c may relocalize Ifit1c within the cytoplasm and stabilize its expression.

### The C-terminal domain of Ifit1c stimulates translation inhibition by Ifit1 and Ifit1b

Next, we sought to determine whether Ifit1c could act as a cofactor for Ifit1 or Ifit1b, by determining translation inhibition activity of Ifit complexes *in vitro*. Increasing concentrations of Ifit1 or Ifit1b were incubated with cap0 or cap1 MHV-Fluc reporter mRNAs (Fig. 9*A*), in a reaction containing RRL with or without the addition of 500 nm MBP-Ifit1c_CTD_. Luciferase signal was measured, as previously, and normalized to the buffer-only or MBP-Ifit1c_CTD_-only condition for each titration series. IC_50_ values from these experiments are given in Table 3.

**Table 3:** **Translation inhibition by Ifit complexes** The values are from the data presented in Fig. 9. The IC_50_ values are the concentrations of Ifit that reduce reporter translation by 50% ± standard error. The data were fitted to [Inhibitor] *versus* normalized response curve (*Y* = 100)/(1 + (*X*^HillSlope^)/(IC_50_^HillSlope^)) using the least-squares method in GraphPad Prism.

Ifit or complex	RNA	IC_50_	*P* value (Ifit only *versus* complex)*^a^*
		*nm Ifit in RRL*	
Ifit1b	cap1-MHV-Fluc	147 ± 27.8	
Ifit1b + MBP-Ifit1c_CTD_ WT	cap1-MHV-Fluc	32.6 ± 11.2	<0.0001
Ifit1b + MBP-Ifit1c_CTD_ YL	cap1-MHV-Fluc	191 ± 28.4	0.6630
Ifit1	cap0-MHV-Fluc	67.9 ± 9.0	
Ifit1 + MBP-Ifit1c_CTD_ WT	cap0-MHV-Fluc	11.4 ± 5.8	<0.0001
Ifit1 + MBP-Ifit1c_CTD_ YL	cap0-MHV-Fluc	68.7 ± 17.4	0.0577
IFIT1	cap0-MHV-Fluc	49.8 ± 7.3	
IFIT1 + MBP-Ifit1c_CTD_ WT	cap0-MHV-Fluc	55.0 ± 7.1	0.5044

*^a^*The curves were compared by extra sum-of-squares F test.

Inclusion of MBP-Ifit1c_CTD_ in the translation reaction decreased the concentration of Ifit1 required to cause a 50% decrease in translation from cap0 MHV-Fluc reporter mRNA by 5-fold (Fig. 9*B*). Similarly, the addition of MBP-Ifit1c_CTD_ decreased the IC_50_ of Ifit1b on cap1 MHV-Fluc mRNA by 5-fold (Fig. 9*C*). MBP-Ifit1c_CTD_ also enhanced translation inhibition by Ifit1b at higher concentrations, allowing almost complete inhibition of translation, suggesting that Ifit1c may promote saturation of RNA binding by Ifit1b. The addition of mutant MBP-Ifit1c_CTD_-YL did not enhance translation inhibition, indicating that interaction between Ifit1 or Ifit1b and Ifit1c is necessary for Ifit1c cofactor activity (Fig. 9, *B* and *C*). When human IFIT3 was added to a translation reaction with human IFIT1, inhibition of cap0-MHV-Fluc translation was enhanced, as we previously described (14). By comparison, the addition of Ifit1c_CTD_ did not affect translation inhibition by human IFIT1 (Fig. 9*D*). Together, these results indicate that Ifit1c can specifically act as a cofactor for both Ifit1 and Ifit1b to enhance their translation inhibition activity.

## Discussion

IFIT proteins play extensive and diverse roles not only in antiviral defense, but also in inflammation, cancer, and autoimmunity (reviewed in Refs. 15, 43, and 44). Studying their function *in vivo* is invaluable for properly understanding their role in modulating such complex diseases. Given the widespread use of animal models, particularly mice, in biomedical research, it is important to understand how their innate immune systems differ from that of humans to properly evaluate the usefulness of data generated by animal studies with regards to human disease. However, in recent years it has become increasingly clear that the human and murine IFIT families differ in key aspects in terms of both their function and their regulation.

Human IFIT1 is well-characterized to bind to cap0-RNA *in vitro* and inhibit its translation (8, 11, 16). As such, human IFIT1 can restrict viruses that produce cap0 mRNA, such as those with mutated 2′-*O*-methyltransferase enzymes (21–23). These viruses are typically attenuated in mouse infection models and in some cases can be partially or fully restored upon knockout of murine Ifit1 (17, 18, 21–23). Human IFIT1 was more recently shown to bind weakly to cap1-RNA and inhibit its translation at high IFIT1 concentrations. As such, human IFIT1 is capable of restricting the replication of viruses with cap1-RNA when expressed at high levels (4, 13). Murine Ifit1, however, does not share this function and can only inhibit cap0-RNA translation (4, 16), a finding that was recapitulated here.

Instead, we found that a related protein, murine Ifit1b, could inhibit the translation of cap1-RNA at nanomolar concentrations but failed to inhibit cap0 or cap2 translation. We show that overexpression of Ifit1b can inhibit the translation of mouse coronavirus model RNAs while restricting viral replication in murine cells. Previously, overexpression of murine Ifit2 was shown to slightly inhibit the replication of both WT and cap0-mutant MHV (17), whereas Ifit2 knockout increased replication of neurotropic MHV and exacerbated viral encephalitis (45). The antiviral effect of Ifit2, however, was due to an enhancement of innate immune signaling, much like we reported recently for murine Ifit1 (32), rather than a direct effect on viral replication. Human IFIT1 can inhibit cap0-mutant strains of a number of human coronaviruses, whereas murine Ifit1 can inhibit cap0-mutant MHV, but neither could inhibit the replication of WT virus (21–23).

This ability of Ifit1b to specifically sense cap1 methylation is quite striking. Previously, IFIT proteins have been identified that can bind to cap1-RNA with low affinity, particularly rabbit IFIT1B, but in all cases could also bind strongly to cap0-RNA (4, 8, 9, 11). Our mutational analysis implicated histidine 192 as a key residue in Ifit1b cap1 sensing, although the exact mechanism of cap1 discrimination remains uncertain. It is possible that specific contacts are made between His^192^ and the RNA 2′-*O*-methyl group itself, to stabilize the Ifit1b-RNA complex, allowing preferential binding to cap1 over cap0. This mechanism of RNA-binding has been shown previously for eIF4E5, one isoform of the cap-binding translation initiation factor eIF4E, from trypanosome parasites. eIF4E5 interacts with cap4-methylated mRNA, a methylation state unique to these parasites, by making specific contacts between certain hydrophobic sidechains and the methylated RNA backbone, resulting in significantly higher affinity of eIF4E5 for cap4- over cap0-RNA (46).

Although the translation of unstructured RNAs was efficiently inhibited by murine Ifit1 and Ifit1b, here it was found that neither protein was capable of inhibiting the translation of a ZIKV reporter mRNA, even at micromolar concentrations. Previously we showed that human IFIT1 effectively inhibited translation of the same ZIKV reporter construct at nanomolar concentrations (14), indicating a fundamental difference in the ability of human IFIT and murine Ifit proteins to bind to the same substrate. It was previously shown that alphaviruses have a very stable stem loop at the immediate 5′ end of the genome, which prevents binding by mouse Ifit1 and confers resistance to type I IFN *in vivo* (47, 48). Destabilizing this RNA secondary structure confers susceptibility to restriction by Ifit1 (47). Flaviviruses, including ZIKV, have a comparable stable stem loop at the very 5′ end of their genomes (49) (Fig. S5), indicating that they, too, may be refractory to binding by murine Ifit proteins for the same reason.

Such differences in the ability of human IFIT1 and murine Ifits to bind RNA with strong 5′ structure may have implications for vaccine development. In a mouse model of West Nile virus (WNV) infection, a *Flavivirus* closely related to ZIKV, Ifit1 knockout did not restore attenuation of a cap0-mutant WNV strain in some tissues and in primary cultures derived from Ifit1 knockout mice (18). Furthermore, even though Ifit1 is expressed throughout the brain following WNV infection (50), WNV infection in the central nervous system was unaffected by Ifit1 knockout (18). However, both murine Ifit1 and human IFIT1 can restrict cap0-mutant WNV replication in certain cell lines (13, 17, 18) Differences in the expression or activity of IFIT proteins in different tissues between humans and mice may have major implications for the safety and efficacy of a cap0 WNV vaccine strain.

Recently, we and others described a functional complex between IFIT1 and IFIT3 in humans, in which IFIT3 acts as a cofactor to stabilize IFIT1 expression and enhance its RNA-binding activity (13, 14). However, in murid rodents, Ifit3 is truncated and lacks the C-terminal domain containing the Y*XXX*L IFIT1 interaction motif and thus cannot interact with Ifit1 (13) (Fig. S9), a finding that was recapitulated here. However, murine Ifit1, Ifit1b, and Ifit1c maintain the Y*XXX*L motif at the C terminus, and we found that these proteins can interact, likely as heterodimers. Like human IFIT complexes, interaction between murine Ifit proteins was shown to increase their thermal stability, suggesting that heterocomplexing is a thermodynamically preferable state. In mouse cells, Ifit1c expression was significantly enhanced by coexpression with Ifit1 or Ifit1b. *In vitro*, the C-terminal domain of Ifit1c was sufficient to enhance translation inhibition by Ifit1 and Ifit1b, similar to the enhancement we observed previously for the human IFIT1-IFIT3 complex (14).

The mechanism by which IFIT cofactors enhance translation inhibition remains to be determined. We previously hypothesized that the long C-terminal domain of human IFIT3, which is extended by two α-helices relative to IFIT1, promoted RNA binding by IFIT1 (14). IFIT3 was shown to make contacts with the C-terminal and pivot domains of IFIT1 (13), which suggested that IFIT3 may alter the flexibility of IFIT1, causing it to remain bound to cap0-RNA and thereby increasing its affinity. This, in turn, allows IFIT1 to more effectively out-compete eIF4E for binding to the mRNA cap, inhibiting translation initiation at lower IFIT concentrations. However, in mice, Ifit1c does not have a long C-terminal tail, which would allow an analogous mechanism of action, suggesting that Ifit1c may act in a different way to enhance RNA binding by Ifit1 and Ifit1b. This may explain why murine Ifit1c could not enhance translation inhibition by human IFIT1 and why human IFIT3 did not bind to murine Ifit1 in a previous study (13).

The Y*XXX*L interaction motif is almost universally conserved in mammalian IFIT1, IFIT1B, and, where applicable, IFIT3 protein sequences (Fig. S10). Given that IFIT1 and IFIT1B are cap-RNA–binding proteins, this proposes a situation in which the IFIT proteins that are capable of binding to capped RNA need to form complexes with regulatory IFIT cofactors. This may allow fine-tuned expression of IFIT proteins that have the potential to inhibit cellular translation (51) and therefore pose a cytotoxic risk. It may also be a way of integrating IFIT-RNA binding into the wider innate immune response. IFIT3 in humans, for example, is known to stimulate innate immune signaling by promoting the interaction between MAVS, an adaptor protein in the cytoplasm downstream of dsRNA sensing and its activating kinase TBK1 (52, 53). The impact of a heterocomplex of IFIT1 and IFIT3, bound to nonself RNA, on innate immune signaling has yet to be investigated. IFIT3 additionally forms a heterocomplex with the proapoptotic IFIT2, via a different interaction interface in the N terminus, and decreases IFIT2-directed cell death (14, 54). Therefore, in humans but not in mice, the regulation of IFIT1 is intrinsically linked to the regulation of IFIT2, because they share a cofactor. The implications of IFIT coregulation in innate immunity, apoptosis, and other cellular processes are still unknown.

In summary, this present study, coupled with previous evolutionary analyses of the IFIT family (3, 4), has revealed convergent mechanisms for RNA binding and complex formation between species, even though the IFIT locus itself has undergone major restructuring. A model for convergent human IFIT and murine Ifit function is presented in Fig. 10. In humans there is a single protein, IFIT1, which binds very strongly to nonself cap0-RNA, but may also weakly bind to self cap1-RNA and inhibit its translation (4, 8, 11, 16). Human IFIT1 is highly expressed in response to IFN (12), along with its cofactor IFIT3, which promotes its stability and translation inhibition activity (13, 14). By contrast in mice, murine Ifit1 binds only to cap0-RNA and is highly expressed (16), whereas murine Ifit1b binds strongly to cap1-RNA but is poorly expressed in stimulated mouse cells, as we have demonstrated here. Both proteins are regulated by Ifit1c, which acts as a cofactor analogous to human IFIT3. In this way, murine and human cells may achieve the same balance of cap0- and cap1-RNA binding during the IFN response. Therefore, considering the IFIT locus together, rather than examining individual genes, could be more valuable in examining IFIT molecular function.

## Experimental procedures

### Plasmids

Ifit promoter sequences were defined as 1 kb upstream of the annotated transcription start site for each Ifit gene, derived from their respective mRNA sequences (Ifit1, NM_008331.3; Ifit1b_1, NM_001362130.1; Ifit1b_2, NM_053217.2; and Ifit1c, NM_001110517.1), except in the case of Ifit1b_2, where only 0.8 kb of sequence was included because of highly repetitive DNA in the most distal 5′ sequence. Promoters were cloned between MluI and XhoI sites in pGL3 Basic (Promega), upstream of firefly luciferase. pRL-TK (Promega) was included as a normalization control.

For reporter RNA transcription, the firefly luciferase reporter gene flanked by the 5′- and 3′-UTRs of the MHV N protein subgenomic mRNA, which shares 5′ and 3′ terminal sequences with all MHV mRNAs (NC_001846.1) was synthesized with a 5′ T7 promoter sequence (IDT) and inserted between EcoRI and PstI sites in pUC57. pUC57-globin-Fluc (14) and pUC57-ZIKV-Fluc (55) were previously described.

For bacterial expression, sequences for murine Ifit1 (NP_032357.2), Ifit1b (NP_444447.1), Ifit1c (NP_001095075.1), Ifit2 (NP_032358.1), Ifit3 (NP_034631.1), and Ifit3b (NP_001005858.2) were inserted into pTriEx1.1 to contain a C-terminal His_8_ tag, as previously described (32). For MBP-tagged proteins, Ifit sequences were inserted between NdeI and BamHI sites in pOPTHM, which contains an N-terminal His_6_ tag followed by an MBP tag. pOPTHM-Ifit1c_CTD_ was generated by PCR amplification of the C-terminal domain of Ifit1c and pOPTH-Ifit1c_CTD_ was generated by overlapping PCR to remove the MBP tag. Mutants were derived from these plasmids by site-directed mutagenesis PCR using overlapping primers. The plasmids for expression of human IFIT1 (10) and IFIT3 (14) have been described.

For mammalian cell expression, pCDNA3.1 containing Ifit sequences with an N-terminal FLAG tag were purchased from Genscript. For coexpression, eGFP was PCR amplified to contain a 5′ NheI site and Kozak sequence and 3′ NdeI and BamHI sites, followed by XbaI. This eGFP fragment was then inserted into pCDNA3.1 between NheI and XbaI sites. Ifit1 or Ifit1b was then inserted between the introduced NdeI and BamHI sites. Ifit1c was PCR amplified to contain a 5′ thosea asigna virus 2A sequence, followed by a FLAG tag. The 3′ end was WT or contained the YL mutation. The 5′ and 3′ sequences were engineered to overlap the regions flanking the pCDNA3.1-eGFP-Ifit1b BamHI site, to allow insertion by Gibson assembly. Since eGFP-Ifit1 did not express in mouse cells, pCDNA3.1-mCherry-Ifit1 was derived from pCDNA3.1-eGFP-Ifit1 by inserting the mCherry tag between NheI and EcoRI sites to replace the eGFP tag. Ifit1c was then inserted into the BamHI site by Gibson assembly, as previously. RNA-binding mutants of Ifit1 (pCDNA3.1-mCherry-Ifit1WM) and Ifit1b (pCDNA3.1-eGFP-Ifit1bWM) were generated by site-directed mutagenesis, producing W144M and W152M mutants, respectively.

### Recombinant protein expression and purification

Recombinant proteins were expressed in Rosetta2 (DE3) pLysS *Escherichia coli* cells (Novagen). The cells were grown to an *A*_600_ of 0.4–1 in 2× TY medium. Protein expression was induced using 1 mm isopropyl β-d-1-thiogalactopyranoside, at 20 °C for 20 h. The cells were harvested and lysed in buffer containing 400 mm KCl, 40 mm Tris-HCl, pH 7.5, 5% glycerol, 2 mm DTT, 0.5 mm phenylmethylsulfonyl fluoride, and 1 mg/ml lysozyme. Proteins were isolated by affinity chromatography on nickel–nitrilotriacetic acid–agarose resin (Qiagen) or PureCube 100 nickel–nitrilotriacetic acid–agarose (Cube Biotech). Proteins were typically polished by FPLC on Superdex 200 increase 10/300 or HiLoad 16/600 columns (GE Healthcare), in buffer I (200 mm KCl, 40 mm Tris-HCl, pH 7.5, 5% glycerol, 1 mm DTT), concentrated to >1 mg/ml, and then stored at −70 °C. Recombinant IFNβ was produced as previously described from HEK293T cells transfected with pCDNA3-IFN-β (56). Supernatant was harvested after 24 h, aliquoted, stored at −70 °C, and diluted 1:500 in cell culture medium to stimulate cells.

### In vitro transcription

Plasmids were linearized with FspI (globin), PmlI (MHV), or HindIII (ZIKV) and purified by gel extraction (globin) or ethanol precipitation (MHV and ZIKV). RNA was transcribed from 0.5 to 2 µg linearized template using 50 ng/μl recombinant T7 RNA polymerase in transcription buffer (40 mm HEPES-NaOH, pH 7.5, 32 mm MgOAc, 40 mm DTT, 2 mm spermidine, 10 mm ATP, 10 mm CTP, 10 mm GTP, 10 mm UTP, 0.2 unit/μl RNaseOUT (Invitrogen)), for 2–4 h at 37 °C. RNA was purified by DNaseI treatment, acidic phenol extraction, and ethanol precipitation. Residual nucleotides were removed using Illustra MicroSpin G-50 columns (GE Healthcare). To produce cap0 and cap1 RNA, 40–60 µg of RNA was capped using the ScripCap m7G capping system and 2′-*O*-methyltransferase system (CellScript). Cap2 RNA was generated from cap1 templates using 200 ng/μl recombinant cap2 methyltransferase in cap2 buffer (50 mm Tris-HCl, pH 7.5, 5 mm DTT, 2 mm SAM, 0.1 unit/μl RNaseOUT) and incubated at 20 ˚C for 4 h before acidic phenol extraction and ethanol precipitation, as described above. Residual nucleotides and SAM were removed using Illustra MicroSpin G-50 columns (GE Healthcare).

Short RNA transcripts (5′-GACATTTGCTTCTGACACAACTGTG-3′) were transcribed from negative-strand DNA oligonucleotide templates, containing a 3′ negative-strand T7 promoter sequence (5′-CACAGTTGTGTCAGAAGCAAA-TGTCTATAGTGAGTCGTATTA-3′), annealed to a T7 promoter-containing forward primer (5′-TAATACGACTCACTATA-3′). RNA was transcribed from 5–10 μm annealed DNA oligonucleotides, in modified transcription buffer (500 ng/μl T7 polymerase, 40 mm HEPES-NaOH, pH 7.5, 13.4 mm MgOAc, 40 mm DTT, 2 mm spermidine, 0.6 mm ATP, 4 mm CTP, 6 mm GTP, 0.6 mm UTP, and 0.1 unit/μl RNaseOUT (Invitrogen)). After purification, to remove residual nucleotides, RNA was polished by FPLC on a HiLoad Superdex 75-pg 16/600 column (GE Healthcare) in MilliQ water at 4 °C, at a flow rate of 1 ml/min. Peak fractions were concentrated by ethanol precipitation. Up to 150 µg (∼ 200 μm) oligonucleotide RNA was capped in modified capping reactions, containing 1 mm (cap0 reactions) or 2 mm (cap1 reactions) SAM (NEB), and residual nucleotides were again removed by size exclusion.

### Denaturing PAGE

Short RNAs were denatured by boiling for 5 min at 75 °C in 50% formamide loading buffer and separated in 15% acrylamide 7 m urea gels (Bio-Rad Mini-PROTEAN format) at 300 V for 35 min. Longer RNAs were separated in 6% acrylamide 7 m urea gels at 300 V for 45 min. The gels were stained in 1× TBE containing 2 µg/ml ethidium bromide for 10 min and then washed twice in water before imaging under 302-nm UV light.

### Primer extension inhibition

For the 2′-*O*-methylation assay, 50 ng of Cy5-labeled primer (Sigma) was annealed to 40 nm RNA by heating to 75 °C for 5 min and snap-cooling on ice. Reverse transcription was carried out using 5 units of AMV reverse transcriptase (Promega) in 20 mm Tris-HCl, pH 7.5, 100 mm KCl, 0.5 mm dNTPs with 0–4 mm MgOAc. For IFIT binding experiments, 25 ng of Cy5-labeled primer was annealed to 10 nm RNA and then incubated with indicated concentrations of IFIT in 20-μl reactions containing 20 mm Tris-HCl, pH 7.5, 100 mm KCl, 2.5 mm MgCl_2_, 1 mm ATP, 0.2 mm GTP, 1 mm DTT, 0.25 mm spermidine, 0.1 unit/μl RNaseOUT, and 0.5 mg/ml BSA. The reactions were incubated at 37 ˚C for 10 min before addition of 2.5 units of AMV reverse transcriptase (Promega), 4 mm MgCl_2_, 0.5 mm dNTPs, and labeled primer, either Cy5 (Fig. S8*A*) or ^32^P (PerkinElmer) (Fig. S8*D*). Reverse transcription reactions were incubated at 37 ˚C for 30 min and then stopped with 100 mm EDTA and 10% SDS. cDNA products were extracted with UltraPure phenol:chloroform:isoamylalcohol (25:24:1), pH 8, (ThermoFisher) and ethanol-precipitated. Pellets were resuspended in 91% formamide loading dye and boiled for 5 min at 75 °C for PAGE. cDNA products were separated by 6% denaturing PAGE on 35-cm sequencing gels for 30–60 min and then imaged directly on an FLA7000 Typhoon scanner (GE Healthcare).

### In vitro translation

For translation inhibition assays, Ifit proteins were serially diluted in BSA buffer in a volume of 2.5 μl (20 mm Tris-HCl, pH 7.5, 150 mm KCl, 5% glycerol, 1 mm DTT, 0.5 mg/ml BSA, 10 units/μl RNaseOUT). 125 ng of Fluc reporter RNA bearing different 5′- and 3′-UTRs (final concentration, 15–20 nm) was added to diluted Ifits and incubated at 30 ˚C for 15 min for RNA binding. *In vitro* translation was then carried out using the Flexi RRL system (Promega) at 30 °C for 90 min. For murine Ifit complexes, 500 nm MBP-Ifit1c_CTD_ or the equivalent volume of buffer I was included in the RRL master mix. The reactions were incubated at 30 ˚C for 90 min and then stopped by the addition of 50 μl of passive lysis buffer (Promega) on ice. Stopped translation reactions were diluted 1:10, and an equal volume of firefly luciferase assay reagent was added, to a final volume of 50 μl. Luminescence was measured by GloMax for 10 s/well. The data were normalized to the no-IFIT control for each experiment. IC_50_ values were derived by fitting to [Inhibitor] *versus* normalized response curve (*Y* = 100)/(1 + (*X*^HillSlope^)/(IC_50_^HillSlope^)) using the least squares method in GraphPad Prism. Confidence intervals were calculated using the likelihood ratio asymmetric method, and a replicates test was performed to test for lack of it. The curves were compared by extra sum of squares F-test.

### Coprecipitation

For coprecipitation experiments, 2.5 μm MBP-tagged bait protein was incubated with 2.5 μm prey protein at 30 ˚C for 1 h in buffer P (20 mm Tris-HCl pH 7.5, 200 mm KCl, 5% glycerol, 0.1% Nonidet P-40, 1 mm EDTA, 5 mm DTT) in a final volume of 40 μl. Proteins were centrifuged at 15,000 × *g* for 60 s to remove any precipitate and then applied to equilibrated amylose magnetic beads (NEB) for 30 min, in a final volume of 200 μl. The beads were washed three times for 1 min in buffer P and then eluted by incubation for 20 min with 100 mm maltose in buffer P. For competition assays, the beads were washed once in buffer P, followed by three washes with increasing concentrations of competitor protein (0.5, 1, and 2 μm), for 10 min each. The beads were washed once in buffer P before elution. 10-μl samples were taken at each stage for SDS-PAGE analysis.

### Differential scanning fluorimetry

To assay RNA binding, a dilution series of 25 nt model RNAs, up to 16 μm, were mixed with 2 μm protein and 1:500 protein thermal shift dye in 20 mm HEPES-NaOH, pH 7.5, 150 mm KCl, 5% glycerol, 1 mm DTT, and 20 ng/μl yeast tRNA (Ambion). For testing the stability of IFIT proteins and complexes, in an optical 96-well reaction plate (Applied Biosystems), 2.5 µg of protein was mixed with 1:500 protein thermal shift dye (Life Technologies) in 20 mm HEPES-NaOH, pH 7.5, 150 mm KCl, 2.5 mm MgOAc, 5% glycerol, and 1 mm DTT, in a final volume of 20 μl. Emission was measured at 623 nm in a ViiA7 real-time PCR system (Applied Biosystems), ramping from 25 to 95 ˚C stepwise at a rate of 1 ˚C per 20S. For interpolation of melting temperatures, the data were analyzed using the Bolzmann equation (*y* = *LL* + (*UL* –*LL*)/(1 + exp(*T*_m_ – x)/1)), where *LL* and *UL* are the minimum and maximum fluorescence intensities, respectively, and melting temperature (*T*_m_) was interpolated from the 50% intersect of the curve.

### Size-exclusion chromatography–multiangle light scattering

Ifit proteins (1 mg/ml) were analyzed by SEC on a Superdex 200 Increase 10/300 GL column at 4 ˚C at a flow rate of 0.5 ml/min. Ifit complexes were examined by combining Ifit proteins at stoichiometric concentrations for 1 h at 4 or 30 ˚C, before SEC analysis. 280-nm absorbance was normalized such that peak height was equal to 1, for ease of comparison. For SEC-MALS, Ifit1b (0.5 or 2 mg/ml in a 150-μl loop) was applied to a Superdex 200 Increase 10/300 GL column at room temperature, at a flow rate of 0.4 ml/min. MALS analysis was performed by inline measurement of static light scattering (DAWN 8+; Wyatt Technology), differential refractive index (Optilan T-rEX; Wyatt Technology), and 280-nm absorbance (Aligent 1260 UV; Aligent Technologies). Molecular mass was calculated using the AS-TRA6 software package (Wyatt Technology). Access to SEC-MALS apparatus was kindly provided by Dr. Janet Deane.

### Cell lines

Human embryonic kidney (HEK293T), murine macrophage-like (RAW264.7) and MEF cell lines all from ATCC were maintained in Dulbecco's modified Eagle's medium (DMEM) with 4.5 mg/ml glucose supplemented with 10% fetal calf serum, 2 mm l-glutamine, penicillin (100 SI units/ml), and streptomycin (100 µg/ml). Murine 17 clone 1 (17Cl-1) cells derived from spontaneously transformed BALB/c 3T3 fibroblasts (30) and kindly shared by Dr. Nerea Irigoyen were maintained in DMEM with 1 mg/ml glucose.

### Transfection of mammalian cells

For Ifit induction experiments, the cells were stimulated with 2 µg of poly(I:C) (Sigma) transfected using Lipofectamine 2000 (Invitrogen). To determine Ifit promoter activity, 17Cl-1 cells were transfected at 70% confluency with 800 ng of pGL3-Ifit promoter plasmid and 200 ng of pRL-TK, using Lipofectamine 2000. After 6 h, the cells were stimulated with 1:100 recombinant IFNβ (produced as described above). The cells were harvested after 24 h by washing in PBS and lysis in passive lysis buffer (Promega). Promoter activity was measured using the Dual-Glo luciferase assay system (Promega) with a Glomax luminometer (Promega). Fluc signal was normalized to Rluc signal, and fold changes were calculated between IFN-treated and mock-treated wells. For puromycylation experiments, 17Cl-1 cells were transfected with 2 µg/well pCDNA3.1-FLAG-Ifit plasmids, or empty pCDNA3.1, using Lipofectamine 2000. After 16 h, nascent proteins were labeled using puromycin at 5 µg/ml for 4 h. The cells were harvested by washing in PBS and lysis in passive lysis buffer. Puromycin signal was detected by immunoblotting, as described below, and was quantified using ImageJ, normalized to the tubulin signal. For Ifit coexpression experiments, 17Cl-1 cells were seeded into 6-well plates containing glass coverslips. At 60% confluency, 2.5 pmol of Ifit coexpression plasmids (described above) were transfected using Lipofectamine 2000. After 24 h, the coverslips were fixed and stained for immunofluorescence microscopy, as described below, and surrounding cells from the same well were harvested for immunoblotting using passive lysis buffer.

### Electroporation of mammalian cells

For infection experiments, transfection using Lipofectamine 2000 inhibited infection, therefore plasmids were transfected by electroporation. 1 × 10^6^ 17Cl-1 cells were mixed with 2.5 pmol of Ifit coexpression plasmids (described above) in 100 μl of Opti-MEM (Gibco), in a 2-mm electroporation cuvette. The cells were electroporated using a NEPA21 electroporator (Nepagene) at 125V for 7.5 s. The cells were seeded subconfluently into 24-well plates with glass coverslips, or into T25 flasks, and left to recover for 18 h before infection.

### Virus culture

Recombinant mouse hepatitis virus strain A59 (MHV-A59) was a gift from Dr. Nerea Irigoyen, derived from a full-length cDNA clone, as described (57, 58). 18 h after plasmid electroporation, the cells were infected at a MOI of 0.1 or 5 pfu/cell in low glucose DMEM containing 50 µg/ml DEAE-dextran and 0.2% BSA. After 45 min at 37 ˚C, inoculum was removed and replaced with fresh medium. After 14 h, the cells on coverslips were fixed and stained for immunofluorescence microscopy, and the cells in T25 flasks were trypsinized, fixed, and stained in suspension for flow cytometry analysis (described below).

### Quantitative PCR

qPCR primers were designed to detect Ifit1b, Ifit1c, Ifit2, Ifit3, and Ifit3b, within the coding sequence of the second exon (Table S1). Primers for Ifit1 have been described (59). End-point PCR was performed using *Taq* polymerase (Invitrogen) on 10 ng of pTriEx1.1-Ifit template plasmid to verify primer specificity. RNA was extracted from cell lysates in passive lysis buffer, using TRI reagent (Sigma), and cDNA was generated using Moloney murine leukemia virus reverse transcriptase (Promega) and random hexamer primers. qPCR was performed using the qPCR core kit for SYBR green I with low ROX passive reference (Eurogentec), with the manufacturer's recommended parameters: 95 ˚C for 15 s and then 60 ˚C for 1 min for 50 cycles. The data were normalized against GAPDH and expressed as fold change over mock (2^−ΔΔ*C*_q_^).

To verify linear amplification, 10-fold serial dilutions of linearized Ifit plasmid were made from 100 ng of (1.5 × 10^10^ copies) to 10 ag (1.5 copies) DNA/well, and qPCR was performed as described above. Linear regression was performed on CT values plotted against log_10_-transformed DNA mass to ensure PCR efficiency was within acceptable parameters (90–110%). To verify target specificity, qPCR products amplified from IFN-treated RAW264.7 cells were purified by gel extraction and Sanger sequenced.

### SDS-PAGE and immunoblotting

The proteins were resolved by 12.5% SDS-PAGE. Where similarly sized proteins were difficult to resolve, the proteins were separated on precast 4–12% NuPAGE Bis-Tris gels (Invitrogen) in MES buffer (Invitrogen) (coprecipitation experiments; Fig. 6), at 180 V for 110 min at 4 °C. The gels were stained using Coomassie Brilliant Blue R, destained in 25% ethanol, and imaged using a Li-Cor Odyssey imaging system. For immunoblotting, separated proteins were transferred to a 0.45-μm nitrocellulose membrane. The membranes were probed with anti-Ifit1 (sc-134949, Santa Cruz, 1:500), anti-IFIT1 (PA3-848, Pierce, 1:500; cross-reactive with murine Ifit1 and Ifit1b), anti-IFIT2 (12604-1-AP, Proteintech, 1:800; cross-reactive against murine Ifit2 and Ifit3/3b), anti-GAPDH (AM4300, Invitrogen, 1:8000), anti-tubulin (ab6160, Abcam, 1:1000), anti-His (34660, Qiagen, 1:1000), anti-FLAG M2-peroxidase (A8592, Sigma, 1:1000), anti-GFP (G1544, Sigma, 1:4000) and anti-mCherry (ab213511 Abcam, 1:1000). The mouse mAb against puromycin was a kind gift from Prof. Ian Goodfellow. Rat polyclonal antibodies against Ifit1b and Ifit1c were raised and purified by Eurogentec. Ifit1b antiserum was raised against CFQMKKATSRENRKRA and ESHKSHIHDSLDELRC peptides and affinity-purified against ESHKSHI-HDSLDELRC. Ifit1c antiserum was raised against CKASNM-QPRGEDRKRA and CEKHIEETLPRISSQP peptides and affinity-purified against CEKHIEETLPRISSQP. The membranes were then probed with IRdye secondary antibodies (Li-Cor) and imaged on an Odyssey CLx imaging system (Li-Cor). For puromycylation experiments, to visualize total protein, the membranes were stained using REVERT (Li-Cor) and then destained, before blocking.

### Immunofluorescence microscopy

The cells were fixed in 3% paraformaldehyde in PBS and then permeabilized in 0.1% Triton X-100 in PBS with 50 mm NH_4_Cl. The cells were blocked in PBS with 0.2% fish gelatin, 0.02% NaN_3_, and 0.01% Triton X-100. The ;Coverslips were stained with anti-FLAG (F1804, Sigma, 1:1000) and anti-dsRNA (10010500, SCIONS English and Scientific consulting, 1:1000) with Alexa Fluor secondary antibodies (Life Technologies), before mounting using ProLong Gold antifade reagent with 4′,6-diamino-2-phenylindole (Invitrogen). Slides were visualized using either the 10× objective (Fig. 4*B*) or the 60× oil immersion objective (Fig. 8, *D* and *E*) of an Olympus IX81 wide field microscope, using Image Pro Plus software. Merged pseudocolored images were generated in ImageJ.

### Flow cytometry

Murine 17Cl-1 cells were electroporated with Ifit expression plasmids, as described above, and then seeded into T25 flasks. After 24 h, the cells (∼2.5 × 10^6^) were infected with MHV A59 at an MOI of 0.05 pfu/cell. After 16 h, the cells were harvested by trypsinization, followed by fixation in 1% paraformaldehyde and permeabilization in ice-cold methanol. The cells were blocked with 2% FCS in PBS, before staining with anti-dsRNA (10010500, SCIONS English and Scientific Consulting, 1:600) and Alexa Fluor secondary antibodies (Life Technologies), before analysis on an Attune NxT flow cytometer (Invitrogen). The data were analyzed using FlowJo (v. 10.6.2).

### Graphs and statistics

The graphs were generated in GraphPad Prism (version 7.03) or Microsoft Excel (Micrsosoft Office 2013, version 15.0.5119.1000). For pairwise comparisons of data means throughout, the data were analyzed by two-tailed Student's *t* test, assuming unequal variance, as indicated in the figure legends. Nonlinear regression was carried out using GraphPad Prism, as described above.

### Structural modeling

RNA secondary structural models and free energy calculations were generated with Mfold (60). Protein homology models were generated using SWISS-MODEL (61), based on known IFIT structures as indicated in the relevant figure legends. Protein structures were visualized using the PyMOL molecular graphics system (version 1.5.0.5, Schrödinger, LLC; RRID:SCR_000305). Structures and models for Fig. 9 were visualized using Illustrate (62).

### Phylogenetic analysis

Mammalian IFIT mRNA sequences were assembled by Daugherty *et al*. (4). Protein sequences were aligned using MUSCLE (63), and maximum likelihood trees were built and visualized in Seaview (64) using PhyML (65), with 100 bootstrap replicates for statistical support. Sequence alignments of IFIT3 proteins in different species were visualized using CIAlign (https://pypi.org/project/cialign/) (66).

## Data availability

All data are contained within the article and the online supporting information.

## Supplementary Material

Supporting Information
