# Supplementary material for: Mouse Ifit1b is a cap1-RNA–binding protein that inhibits mouse coronavirus translation and is regulated by complexing with Ifit1c
Source: J Biol Chem. 2020 Oct 19;295(51):17781–801. doi: 10.1074/jbc.RA120.014695 (PMC7762956; doi:10.1074/jbc.RA120.014695)
Supplement: Supporting Information [file supp_295_51_17781__index.html]

Mouse Ifit1b is a cap1-RNA binding protein which inhibits mouse coronavirus translation and is regulated by complexing with Ifit1c — Mouse Ifit1b paralogues are functional antiviral effectors — Mouse Ifit1b is a cap1-RNA–binding protein that inhibits mouse coronavirus translation and is regulated by complexing with Ifit1c — Mouse Ifit1b paralogues are functional antiviral effectors — Supporting Information 

# Mouse Ifit1b is a cap1-RNA–binding protein that inhibits mouse coronavirus translation and is regulated by complexing with Ifit1c

## Supporting Information

- Supporting Information (to be published online) - Supporting information including Figures S1-S13 and Table S1
