# Supplementary material for: Mouse Ifit1b is a cap1-RNA–binding protein that inhibits mouse coronavirus translation and is regulated by complexing with Ifit1c
Source: J Biol Chem. 2020 Oct 19;295(51):17781–801. doi: 10.1074/jbc.RA120.014695 (PMC7762956; doi:10.1074/jbc.RA120.014695)
Supplement: Supporting Information [file supp_RA120.014695_161323_1_supp_608693_qhvvvz.pdf]

Supporting Information for:

Mouse Ifit1b is a cap1-RNA binding protein which inhibits mouse coronavirus translation and is regulated by complexing with Ifit1c

Harriet V. Mears[1*] and Trevor R. Sweeney[1*]

1 Division of Virology, Department of Pathology, University of Cambridge, Addenbrooke's Hospital, Hills Road, Cambridge, UK.

* Co-corresponding authors: Harriet Mears and Trevor Sweeney
Email: hvm30@cam.ac.uk and ts629@cam.ac.uk

Contains Supplementary Figures S1-S13 and Table S1.

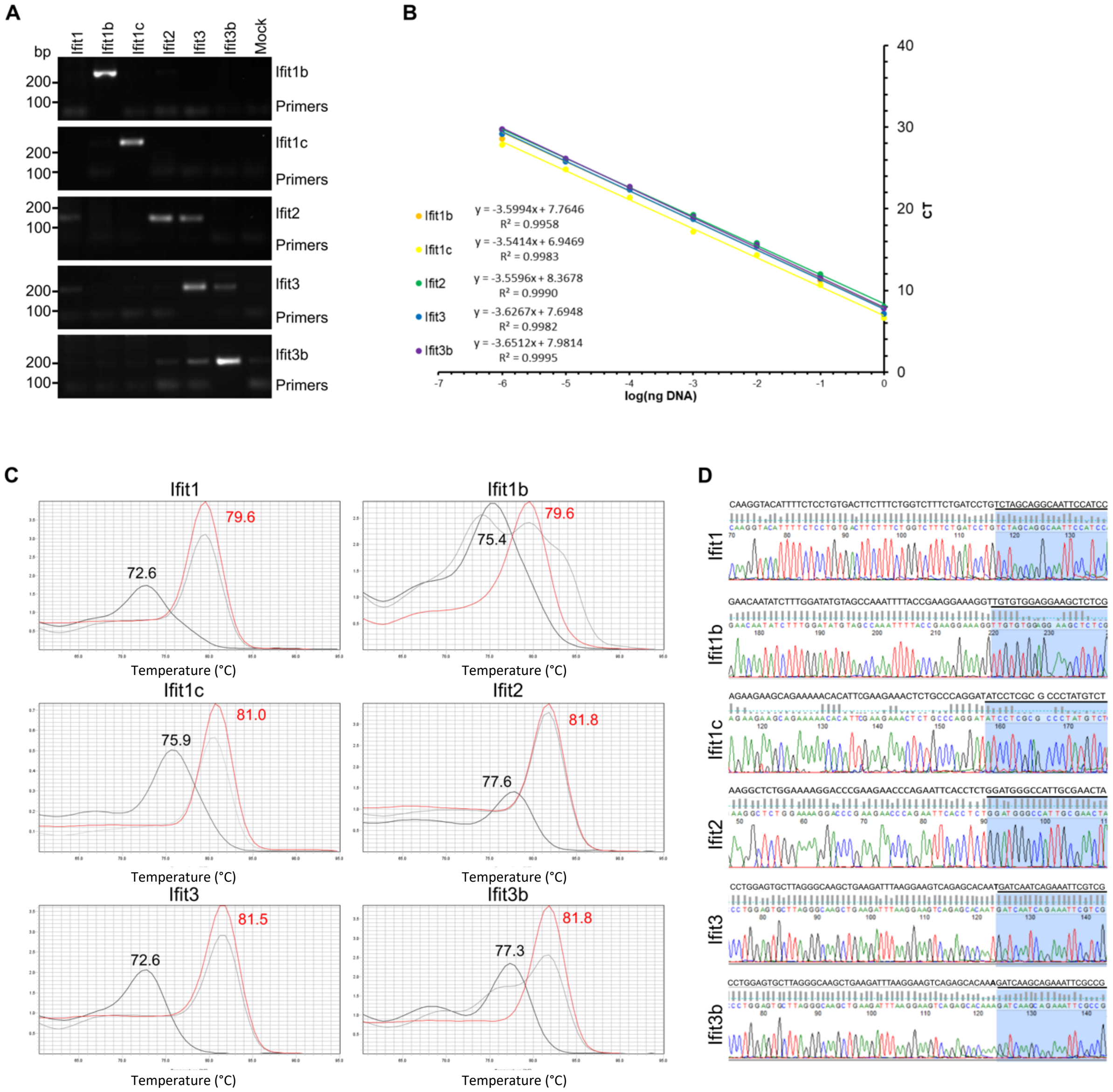

Figure S1. Validation of qPCR primer pairs for detection of murine Ifit mRNA.

(A) Ifit qPCR primer pairs were tested for specificity using plasmids containing the coding sequence of each Ifit gene or water (Mock) as indicated. (B) Linear amplification was tested by ten-fold serial dilution of linearised plasmid DNA templates. Log_{10}-transformed mass of DNA (ng) was plotted against qPCR cycle threshold (CT) and fit by linear regression. Equations and R^2 values showing linearity and goodness of fit are given next to the graph legend. (C) Melt curves from qPCR amplification of cDNA from RAW264.7 cells, either mock-treated (grey) or treated with type I IFN (red), or water (black). Melting temperatures are given. (D) Sanger sequencing analysis of RT-qPCR products from stimulated RAW264.7 cells, with primer sequences highlighted in blue. Reference sequences are given above and primer sequences are underlined.

Table S1. qPCR primer sequences.

Target	Forward primer	Reverse primer
Ifit1	5'-CCAAGTGTTCCAATGCTCCT-3'	5'-GGATGGAATTGCCTGCTAGA-3'
Ifit1b	5'-AACCCTGAGTACAACGCTGG-3'	5'-CGAGAGCTTCCTCCACACAA-3'
Ifit1c	5'-CAATGCTGGCTATGCAGTCG-3'	5'-AGACATAGGGCTGCGAGGAT-3'
Ifit2	5'-CTTGACTGTGAGGAGGGGTG-3'	5'-TAGTTCGCAATGGCCCATCC-3'
Ifit3	5'-AGACAGGGTGTGCAACCAGG-3'	5'-CGACGAATTTCTGATTGATC-3'
Ifit3b	5'-AGACAGGGTGTGCAACCAGC-3'	5'-CGGCGAATTTCTGCTTGATC-3'
Viperin	5'-GGTTCAAGGACTATGGGGAG TATTTGGAC-3'	5'-GAAATCTTTCTGCTTCCCTCA GGGCATC-3'
GAPDH	5'-CATGGCCTTCCGTGGTTCCTA-3'	5'-GCGGCACGTCAGATCCA-3'

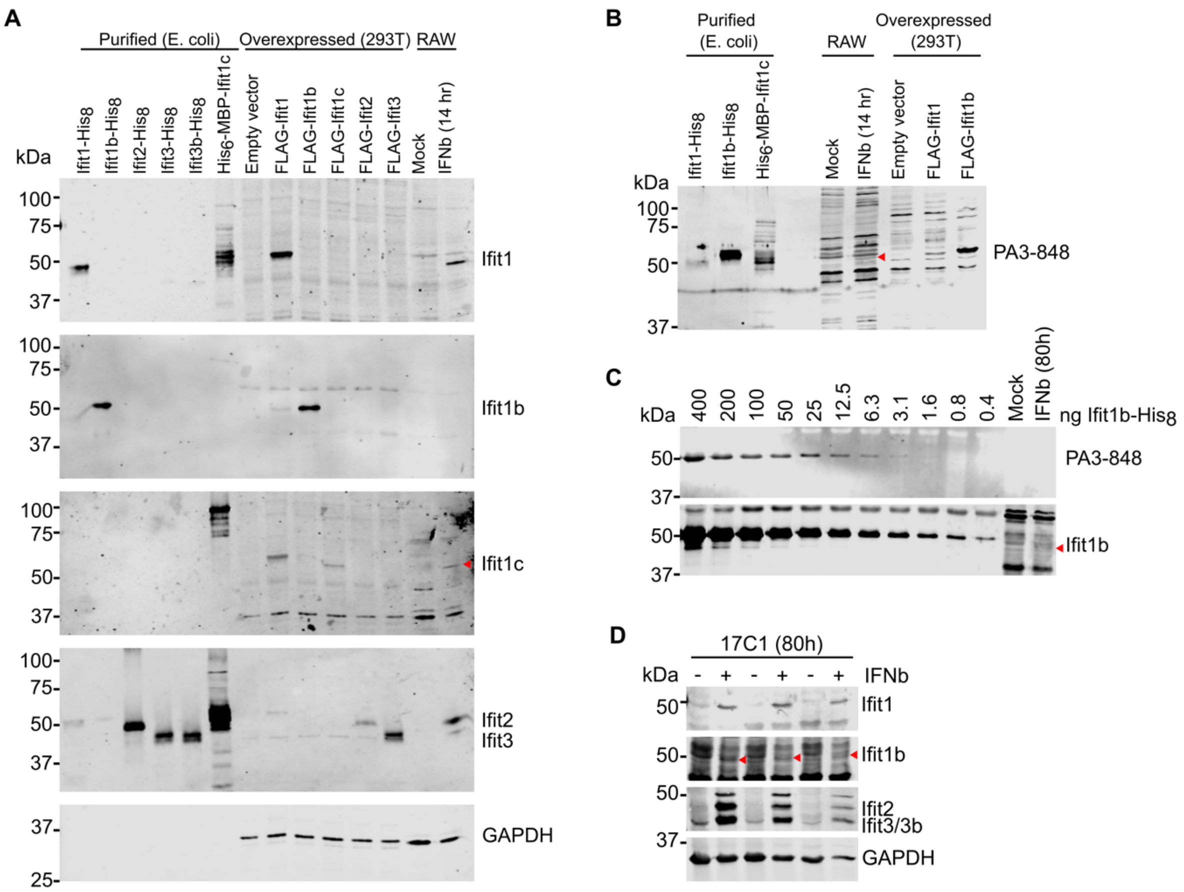

Figure S2. Validation of antibodies for detection of murine Ifit proteins.

(A) Western blotting of RAW264.7 cells stimulated with IFNβ for 14 hours alongside recombinant Ifit proteins purified from E. coli or overexpressed in HEK293T cells, probed with antibodies against Ifit1 (Santa-cruz, sc-134949); Ifit1b, using a peptide-raised antibody (Eurogentec); and Ifit1c, using a peptide raised antibody (Eurogentec); Ifit2 and Ifit3, using a cross-reactive antibody for human IFIT2 (Proteintech, 12604-1-AP). GAPDH is included as a loading control for cell lysates. (B) The specificity of PA3-848, which detects human IFIT1, (Pierce, anti-IFIT1) was tested against recombinant proteins and cell lysates as described for panel (A). A band is visible in IFN-stimulated RAW264.7 cell lysates, indicated by a red arrowhead. (C) Serial dilution of recombinant Ifit1b protein, to test detection limit of Ifit1b antibodies. In the lower panel, bovine serum albumin, used to ensure accurate serial dilution, is faintly visible. IFN-stimulated or mock-treated 17Cl-1 cell lysates were run alongside. Ifit1b is visible in IFN-treated cells, and runs slightly smaller than recombinant Ifit1b, owing to the additional molecular weight of the His-8 tag. (D) 17Cl-1 cells were stimulated in triplicate with IFNβ and harvested after 80 hours, before immunoblot analysis.

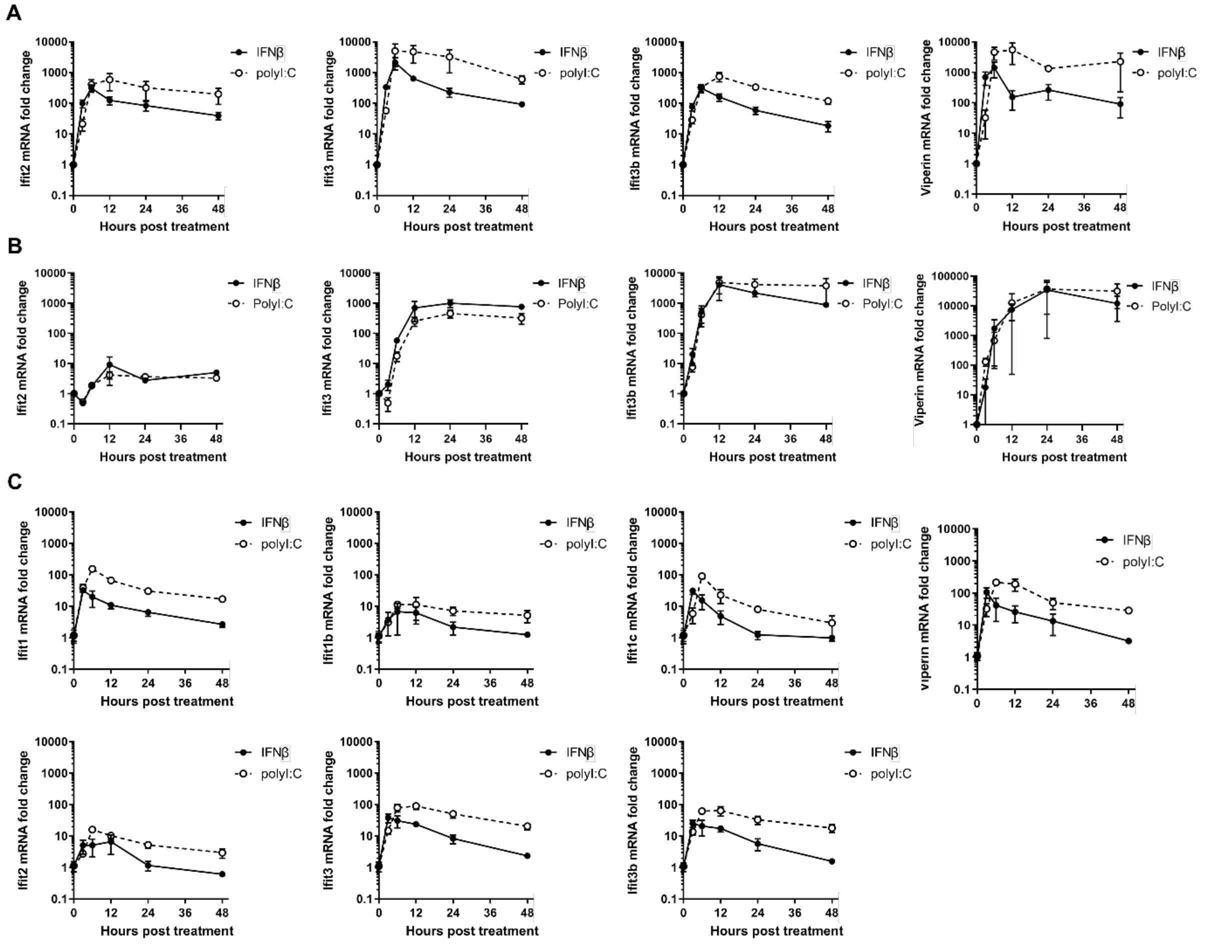

Figure S3. Ifit expression in stimulated murine cells.

(A-B) qPCR analysis of Ifit2, Ifit3 and Ifit3b expression in stimulated (A) RAW264.7 or (B) 17Cl-1 cells, from experiments in Figure 1B-C. Viperin is included as a positive control. (C) qPCR analysis of Ifit and Viperin expression in stimulated murine embryonic fibroblasts. Graphs show the mean and standard error of two biological replicates.

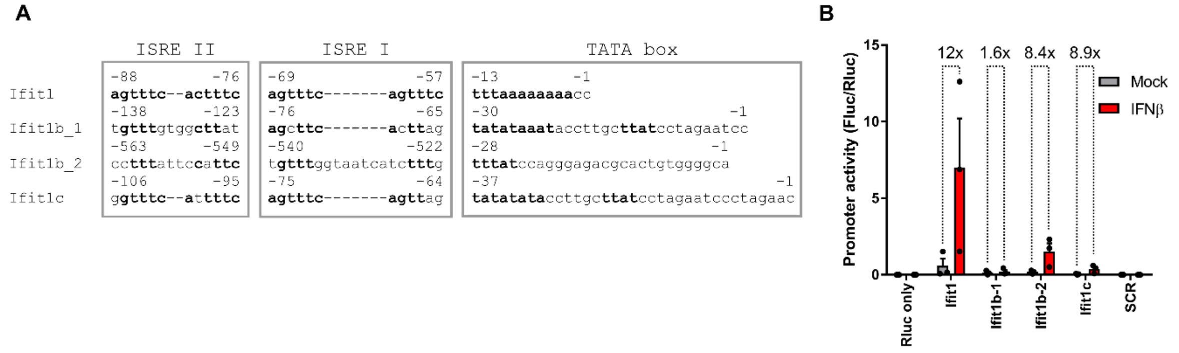

Figure S4. Activity of mouse Ifit promoter sequences.
(A) Annotation of the known (Ifit1) and putative (Ifit1b and Ifit1c) ISRE elements upstream of the Ifit gene transcription start sites. (B) Promoter-driven firefly luciferase (Fluc) activity in mock or IFNβ-stimulated 17Cl-1 cells, normalised to constitutive Renilla luciferase (Rluc) activity. Graph shows the mean and standard error of three biological repeats. A scrambled (SCR) DNA sequence is included as a negative control.

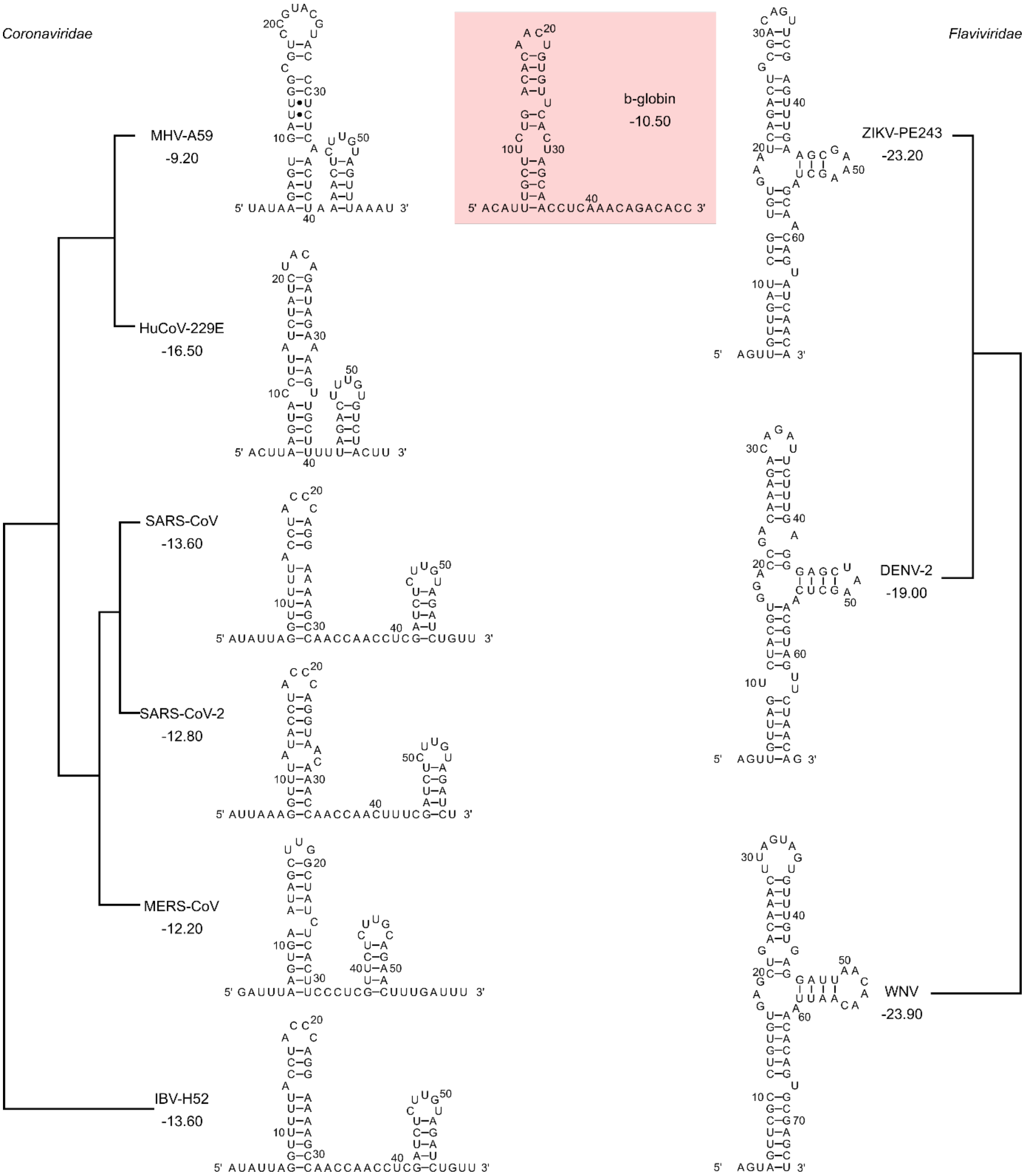

Figure S5. Conserved coronavirus and flavivirus 5′ terminal RNA structural elements.
RNA structures and models of different cornavirus and flavivirus species (mouse hepatitis virus strain A59, MHV-A59; Human coronavirus 229E, HuCoV-229E; severe acute respiratory syndrome coronavirus, SARS-CoV; severe acute respiratory syndrome coronavirus 2, SARS-CoV-2; middle east respiratory syndrome coronavirus, MERS-CoV; infectious bronchitis virus strain H52, IBV-H52; Zika virus strain PE243, ZIKV-PE243; Dengue virus serotype 2, DENV-2; West Nile virus, WNV). The 5′UTR of human β-globin is shown for comparison. Gibbs free energies (*ΔG*, kJ/mol) calculated in Mfold are shown below each virus name. Trees represent the relatedness of the indicated viruses.

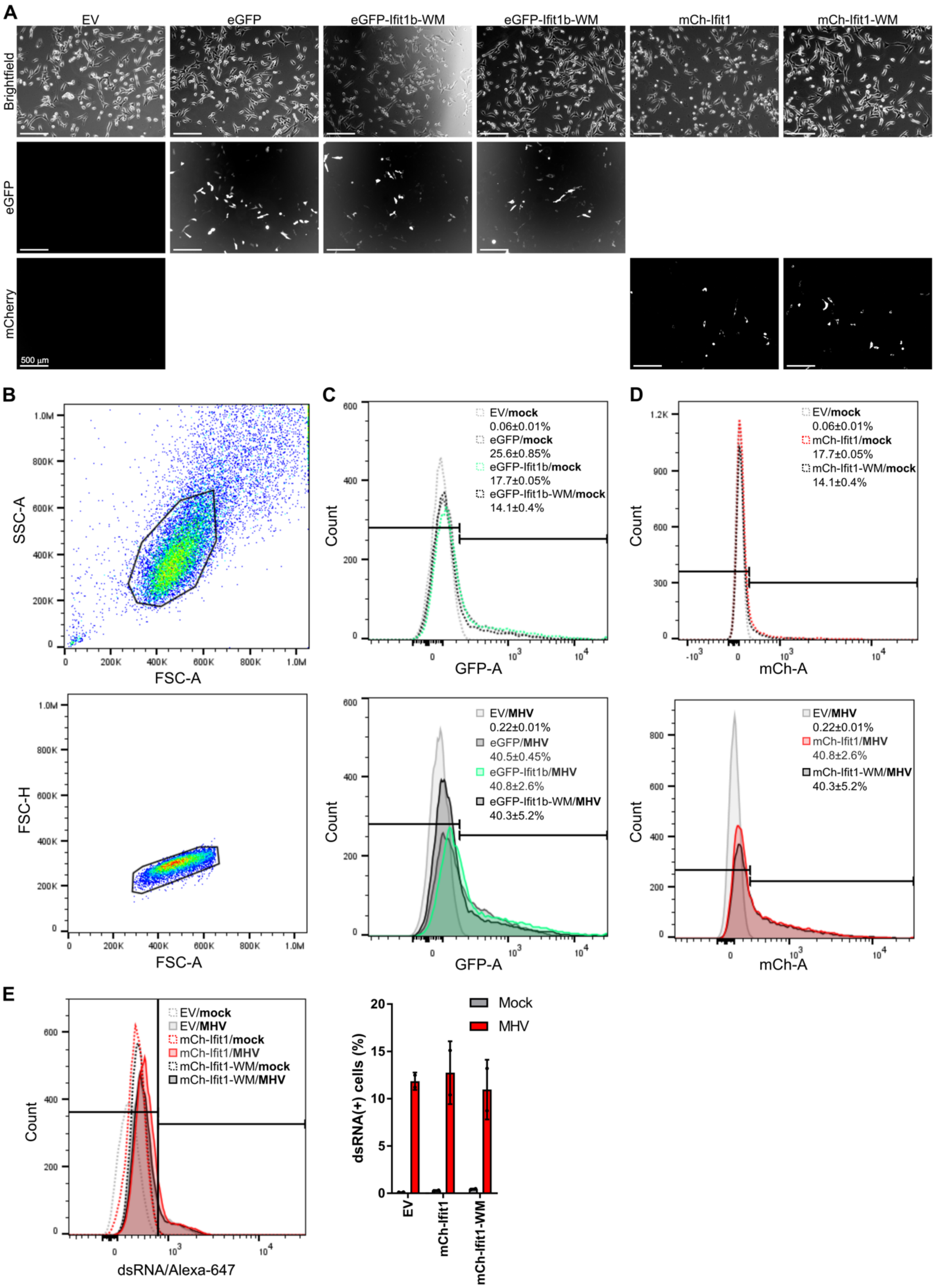

Figure S6. Flow cytometry gating strategy and protein expression levels.

(**A**) Overexpression in 17Cl-1 cells prior to infection with MHV. Cells were electroporated with the indicated plasmids and expression was checked by fluorescence microscopy after 20 hours to ensure expression was equivalent before proceeding with infection experiments. (**B**) Gating strategy for flow cytometry analysis of cells (upper panel) and singlets (lower panel) in cell populations from experiments

described in Figure 4. Mock-infected, empty vector-transfected cells are shown as an example. FSC, forward scatter; SSC, side scatter; A, signal area; H, signal height. (**C**) GFP or (**D**) mCherry expression in transfected cells, either mock (upper panel, dashed lines) or infected with MHV (lower panel, solid lines with shading). Histograms are representative of two independent experiments. (**E**) Flow cytometry analysis of mock (dashed lines) or MHV-infected (solid lines with shading) cells transfected with empty vector (EV, grey), mCherry-Ifit1 (red) or mCherry-Ifit1-WM, an RNA-binding mutant (black). The black vertical line indicates the gate for dsRNA-positive cells on the x-axis. Quantification from this gate is shown in the right panel. Data represent the mean and standard deviation from two independent experiments.

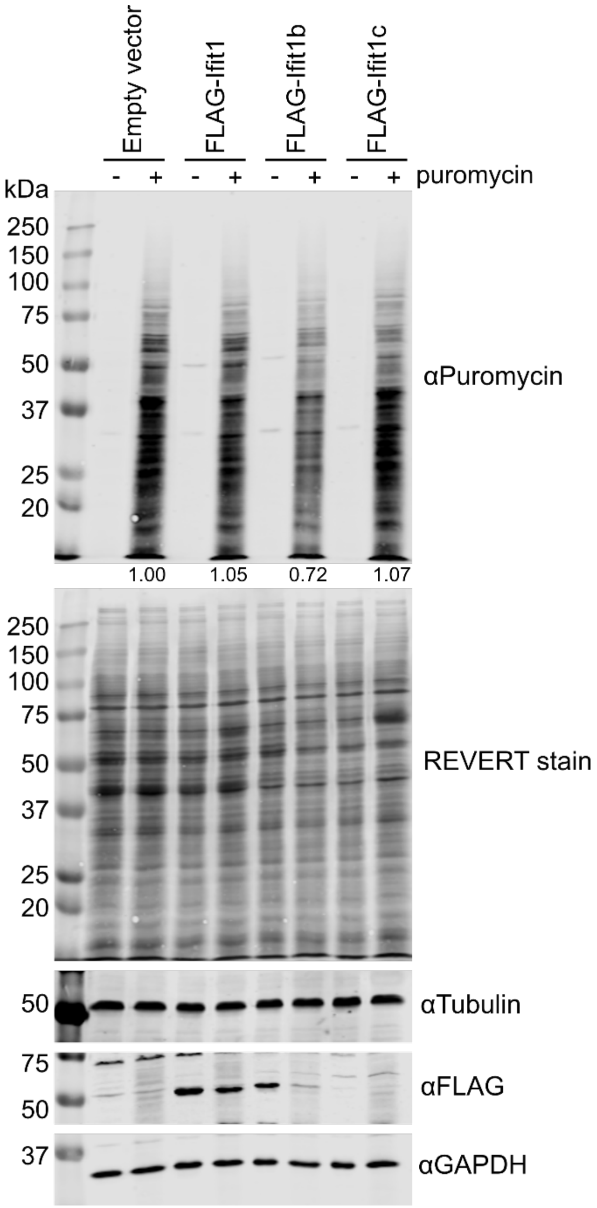

Figure S7. Inhibition of host cell translation by murine Ifit1b.

17Cl-1 cells were transfected with the indicated Ifit expression plasmids. After 18 hours, cells were treated with 5 µg/mL puromycin for a further 4 hours, before cell lysates were harvested and analysed by immunoblotting. Membranes were stained with REVERT total protein stain, before blocking, to show even loading. Tubulin is included as an additional loading control. The puromycin signal was quantified in ImageJ, normalised to the tubulin signal and expressed as a fold change over the empty vector control below each lane in the top panel. Representative of two separate experiments.

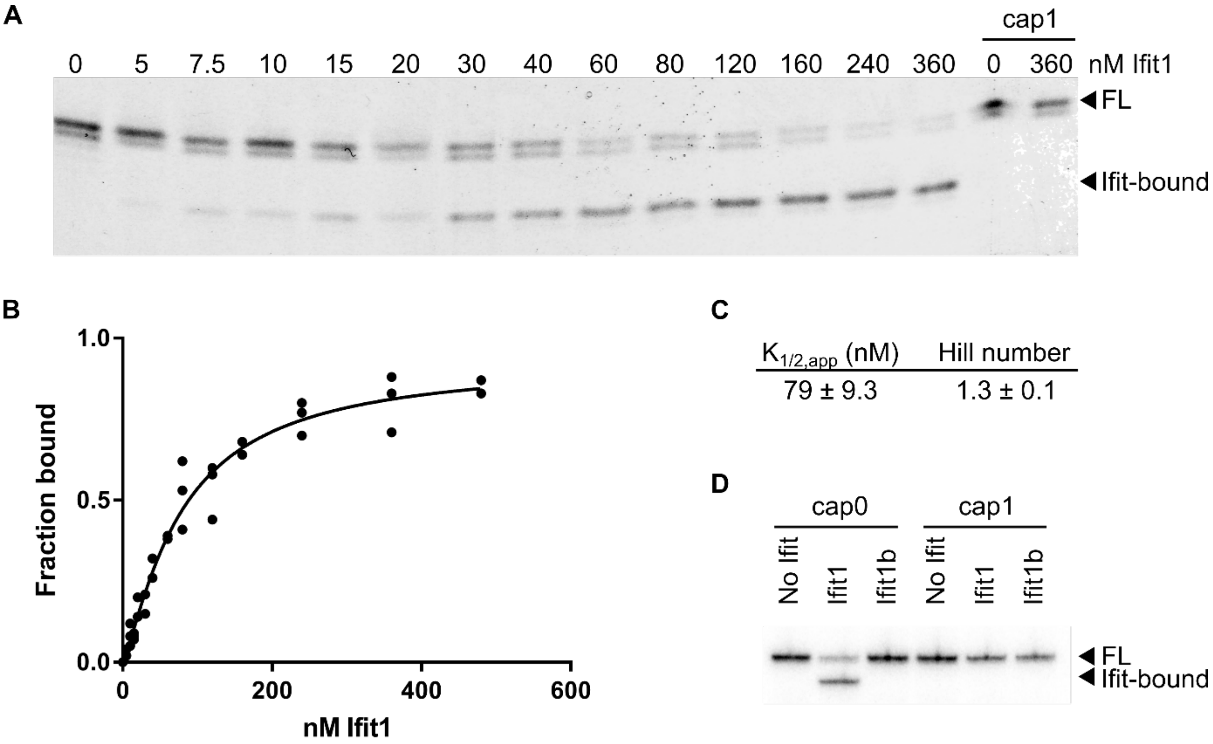

Figure S8. Analysis of RNA binding by primer extension inhibition.

(A) Representative gel showing primer extension analysis of cap0 Globin-Fluc RNA incubated with increasing concentrations of Ifit1. Toeprints induced by Ifit-RNA interaction are indicated. (B) Plot of Ifit1-cap0-RNA interaction. Data were fitted to the non-linear Hill equation ($\text{Fraction}^{[\text{bound}]} = [\text{IFIT1}]^{h} \bullet \text{Fraction}^{[\text{bound}]}_{\text{max}}/([\text{IFIT1}]^{h} + K^{h}_{1/2,\text{app}})$ from data where [IFIT1] was $\geq 10 \bullet$ [mRNA]) using GraphPad Prism. (C) The dissociation constant ($K_{1/2\text{app}}$) and Hill coefficient (h) were calculated from (B). (D) Primer extension analysis of cap0 or cap1 Globin-Fluc RNA incubated with 500 nM Ifit1 or Ifit1b.

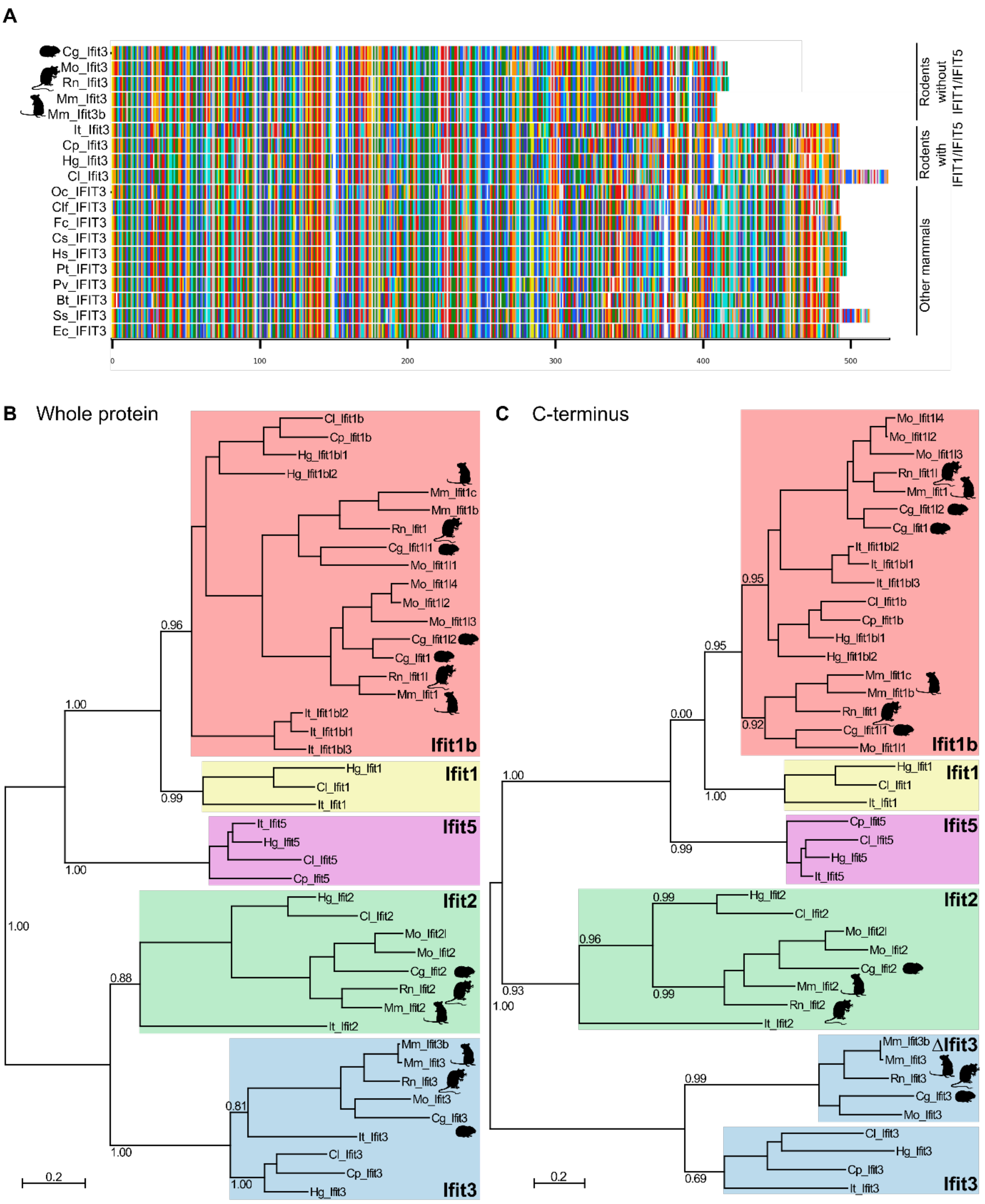

Figure S9. Alignment of mammalian IFIT3 sequences.

(A) Sequence alignment of mammalian IFIT3 sequences, generated in MUSCLE and visualised using CIAlign. (B,C) Maximum-likelihood phylogenetic trees were constructed in PhyML using (B) whole protein or (C) C-terminal sequence alignments (4) of Ifits from eight different rodent species (Cg, Chinese hamster; Cl, chinchilla; Cp, guinea pig; Hg, naked mole rat; It, thirteen-lined ground squirrel; Mm, house mouse; Mo, prairie vole; Rn, Norway rat). Bootstrap supports are shown for major branches. Scale bars represent amino acid substitutions per position. Note that species in which Ifit1 and Ifit5 have been lost, Ifit3 is also truncated.

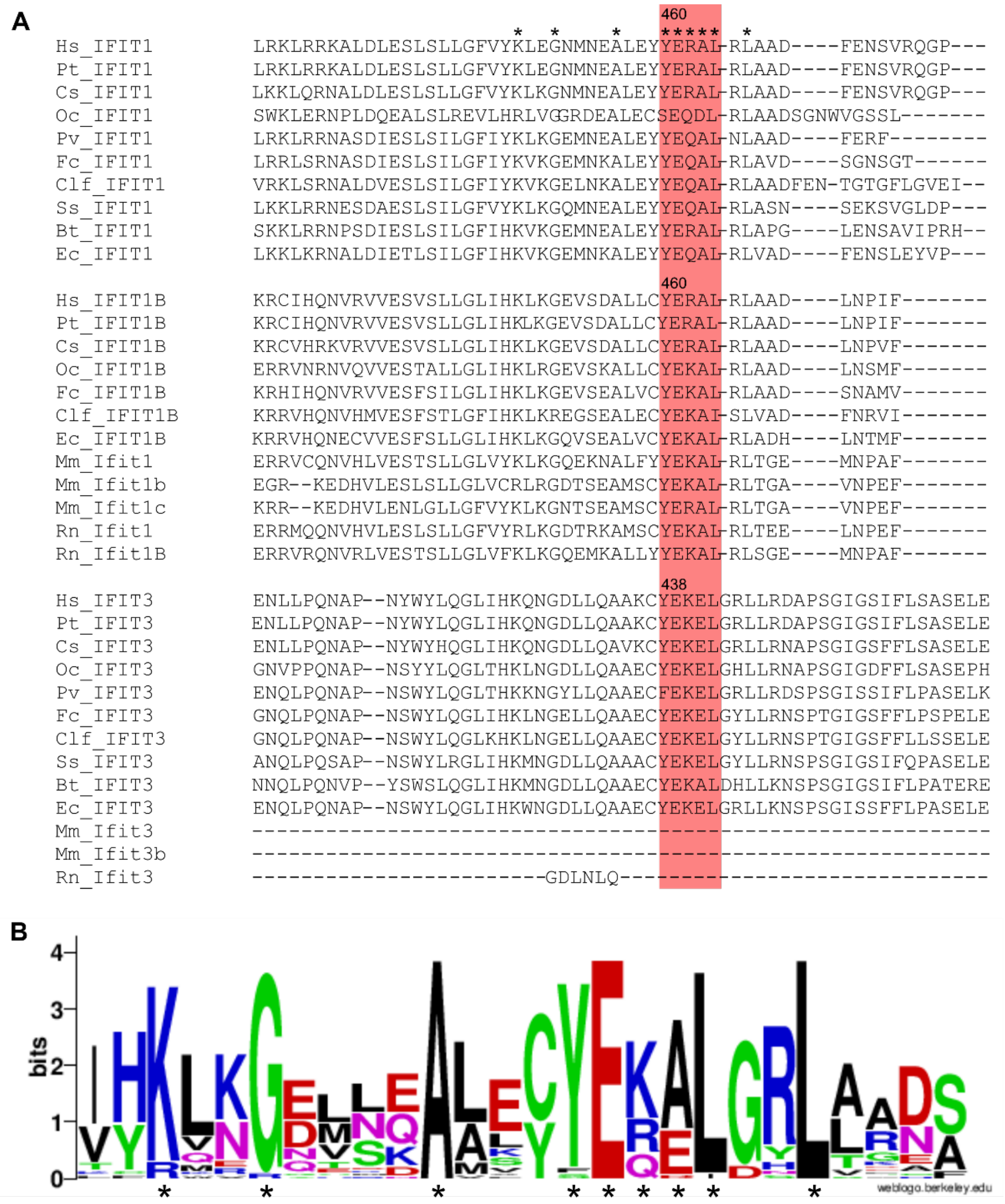

Figure S10. Conservation of YxxxL motif within IFIT1, IFIT1B and IFIT3 proteins from representative mammals.

(A) Sequence alignment showing a portion of the C-terminal domain of IFIT1, IFIT1B and IFIT3 proteins from different species of mammals (Hs, human; Pt, chimpanzee; Cs, African green monkey; Oc, rabbit; Pv, flying fox; Fc, cat; Clf, dog; Ss, pig; Bt, cow; Ec, horse; Mm, mouse; Rn, rat). The conserved YxxxL motif is highlighted in red. (B) Sequence logo showing the conservation of the YxxxL motif and surrounding residues. Asterisks indicate highly conserved residues.

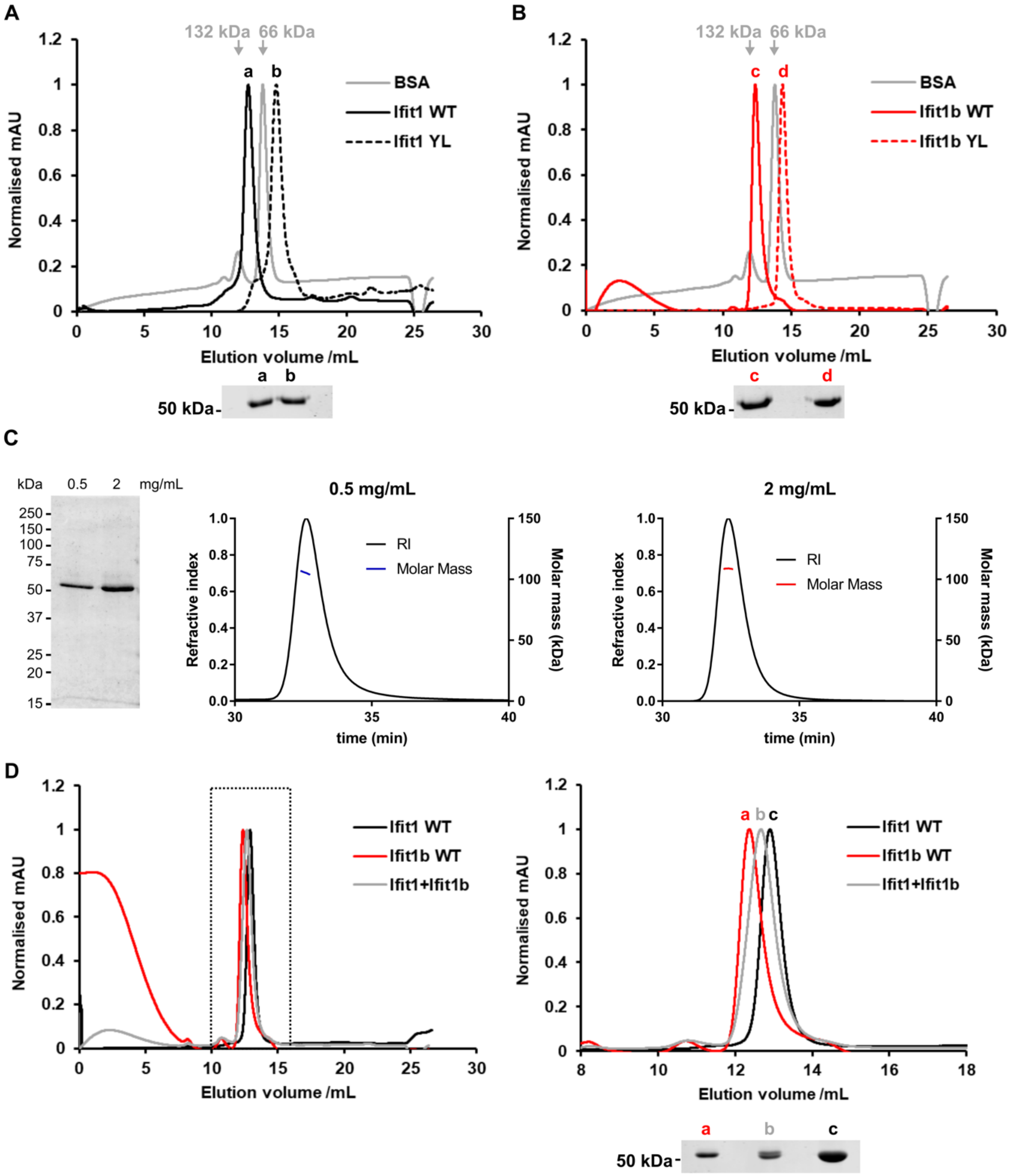

Figure S11. SEC and SEC-MALS analysis of Ifit proteins.

(A-B) Purified wildtype (WT) and YL mutant (A) Ifit1 and (B) Ifit1b proteins were injected onto a Superdex200 Increase 10/300 size exclusion column at 1 mg/mL in a 150 µL loop. Bovine serum albumin (BSA) is shown as a size marker on both graphs (grey line), and the molecular weight of the dimeric and monomeric BSA species are shown above. Gel inserts show SDS-PAGE analysis of proteins eluting in each peak, annotated with lower case letters. (C) Purified WT Ifit1b was injected onto a Superdex200 Increase 10/300 size exclusion column at 0.5 mg/mL or 2 mg/mL, and the mass of eluting species was monitored by MALS. Input proteins are shown in the SDS-PAGE panel on the left. Black lines represent the normalised refractive index of proteins eluting from the column, shown on the left axis, while coloured lines represent the molar mass of the peaks, shown on the right axis. (D)

Purified WT Ifit1 or Ifit1b alone or incubated together for 1 hour at 4 °C were injected onto a Superdex200 Increase 10/300 column at 1 mg/mL. The right panel shows a zoomed in version of the dashed box.

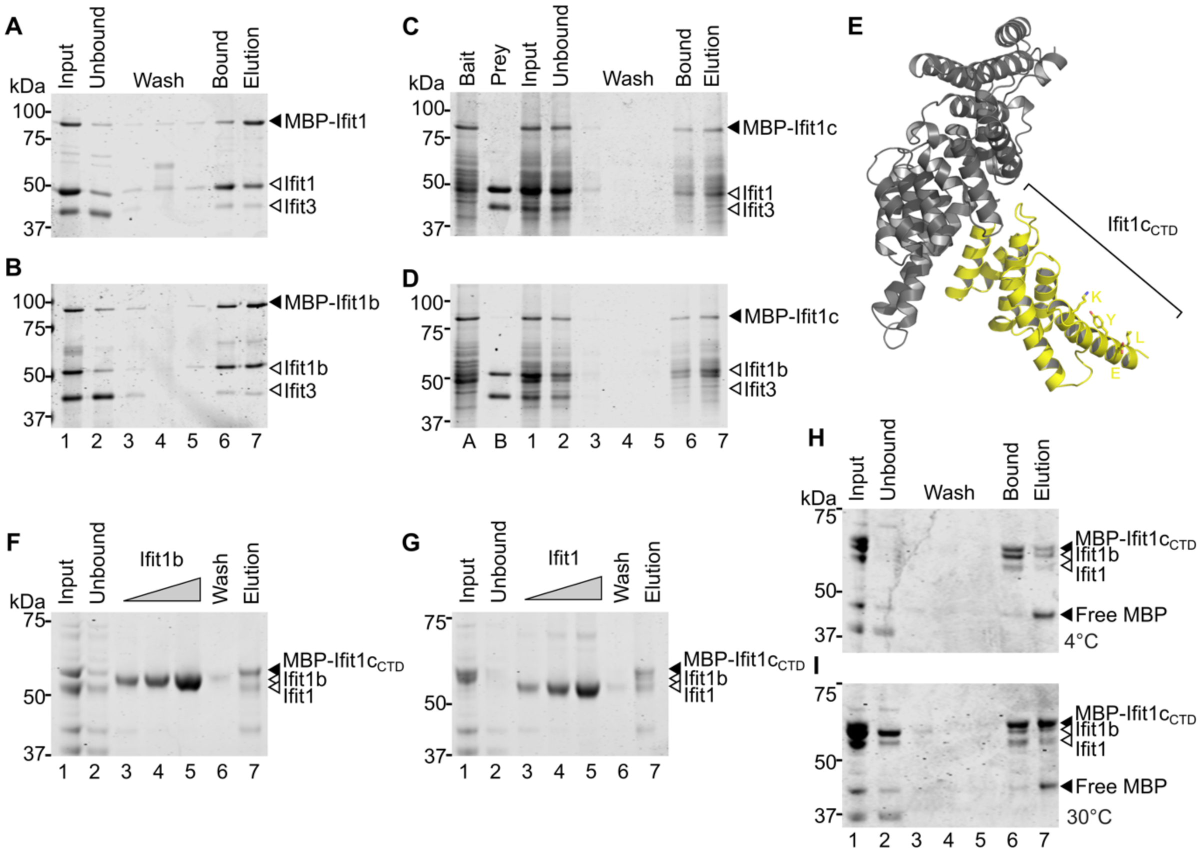

Figure S12. Ifit heterocomplexes.

(A-D) Co-precipitation of (A) Ifit1 with MBP-tagged Ifit1, (B) MBP-tagged Ifit1b with Ifit1b, (C) MBP-tagged full-length Ifit1c with Ifit1, or (D) MBP-tagged full-length Ifit1c with Ifit1b. MBP-tagged bait was incubated with prey proteins (lane 1) before binding to amylose resin. Unbound proteins were washed away (lanes 2-5) and bound proteins remained on the beads (lane 6). Bound proteins were eluted in maltose-containing buffer (lane 7). Ifit3 was included as a negative control in each experiment. In (C and D), for clarity the bait (lane A) and prey (lane B) were run alongside the input sample. (E) Model of murine Ifit1c, based on the structure of human IFIT1 (PDB: 5W5H) generated in SWISS-MODEL, showing the C-terminal domain (CTD) in yellow. (F,G) Competitive co-precipitation of Ifit1, Ifit1b and MBP-tagged $Ifit1c_{CTD}$. MBP-tagged bait ($Ifit1c_{CTD}$) was incubated with (F) Ifit1 or (G) Ifit1b (lane 1) before binding to amylose resin. Unbound proteins were washed away (lane 2) and resin was washed with an increasing concentration of the indicated competitor prey protein (lanes 3-5). Resin was washed again (lane 6) before elution in maltose-containing buffer (lane 7). (H,I) Co-precipitation of Ifit1, Ifit1b and MBP-$Ifit1c_{CTD}$ (lane 1), incubated together at (H) 4 ˚C or (I) 30 ˚C before binding to amylose resin. Unbound proteins were washed away (lanes 2-5) and bound proteins remained on the beads (lane 6). Bound proteins were eluted in maltose-containing buffer (lane 7).

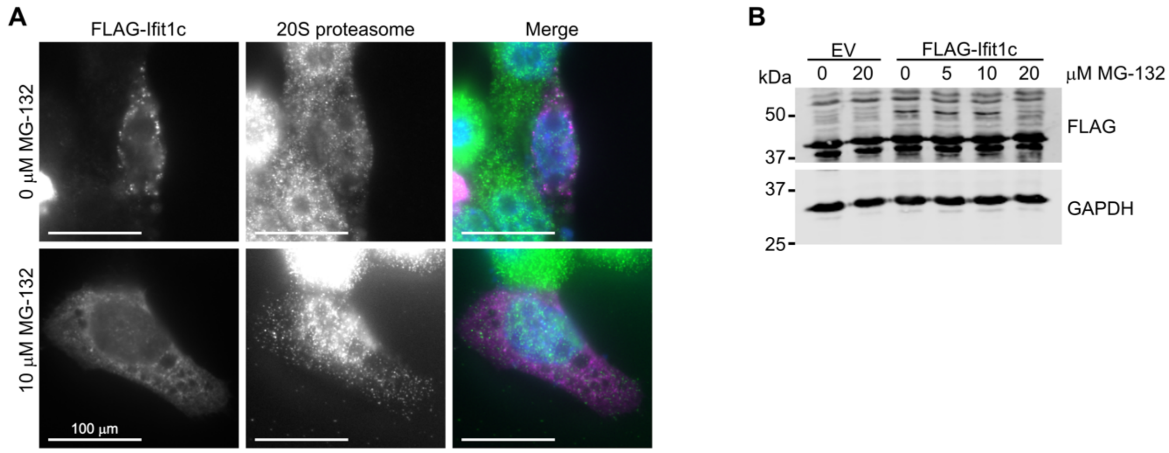

Figure S13. Effect of MG-132 treatment on Ifit1c expression and localisation.
(A) Immunofluorescence microscopy of FLAG-Ifit1-transfected 17Cl-1 cells with or without MG-132 treatment. (B) Western blot analysis of empty vector (EV) or FLAG-Ifit1c-transfected 17Cl-1 cells with increasing concentrations of MG-132. Less FLAG-Ifit1c was detected at 20 μM MG-132, likely due to cytotoxicity of MG-132 at high concentrations.
